# Polymorphic α-Synuclein Strains Modified by Dopamine and Docosahexaenoic Acid Interact Differentially with Tau Protein

**DOI:** 10.1007/s12035-020-01913-6

**Published:** 2020-04-29

**Authors:** Urmi Sengupta, Nicha Puangmalai, Nemil Bhatt, Stephanie Garcia, Yingxin Zhao, Rakez Kayed

**Affiliations:** 1 Mitchell Center for Neurodegenerative Diseases, University of Texas Medical Branch, Galveston, USA; 2 Departments of Neurology, Neuroscience and Cell Biology, University of Texas Medical Branch, Galveston, USA; 3 Department of Internal Medicine, University of Texas Medical Branch, Galveston, USA; 4 Institute for Translational Sciences, University of Texas Medical Branch, Galveston, USA

**Keywords:** α-Syn oligomeric strains, Seeding, Internalization, Cross-seeding, Aggregated tau strains

## Abstract

The pathological hallmark of synucleinopathies, including Parkinson’s disease (PD), is the aggregation of α-synuclein (α-Syn) protein. Even so, tau protein pathology is abundantly found in these diseases. Both α-Syn and tau can exist as polymorphic aggregates, a phenomenon that has been widely studied, mostly in their fibrillar assemblies. We have previously discovered that in addition to α-Syn oligomers, oligomeric tau is also present in the brain tissues of patients with PD and dementia with Lewy bodies (DLB). However, the effect of interaction between polymorphic α-Syn oligomers and tau has not been scrupulously studied. Here, we have explored the structural and functional diversity of distinct α-Syn oligomers, prepared by modifying the protein with dopamine (DA) and docosahexaenoic acid (DHA). The two α-Syn oligomers differed in aggregate size, conformation, sensitivity to proteinase K digestion, tryptic digestion, and toxicity, suggesting them as distinct α-Syn oligomeric strains. We examined their internalization mechanisms in primary neurons and seeding propensity in inducing α-Syn aggregation. Using a combined approach of molecular and cellular techniques, we observed that the tau aggregates cross-seeded with the individual α-Syn oligomeric strains differed in their biochemical and biological properties, suggesting two distinct tau strains. The tau aggregate cross-seeded with the DA-modified α-Syn oligomeric strain possessed a potent intracellular tau seeding propensity. This study provides a comprehensive analysis of unique strain-specific interaction between oligomeric α-Syn and tau. Furthermore, this study allows us to speculate that distinct α-Syn-tau interactions inducing tau aggregation might be an underlying mechanism of neurodegeneration in PD.

**Electronic supplementary material:**

The online version of this article (10.1007/s12035-020-01913-6) contains supplementary material, which is available to authorized users.

## Introduction

Synucleinopathies consist of multiple neurodegenerative diseases, among which Parkinson’s disease (PD) is one of the three major synucleinopathies [1–3]. PD is considered as the second most common form of neurodegenerative disease. Aggregates of α-synuclein (α-Syn) are the primary causative protein aggregates that are recognized as the pathological hallmarks for synucleinopathies, including PD. α-Syn is primarily a presynaptic protein that is abundantly expressed throughout the brain [4]. Several studies have shown that amyloid oligomers can be formed by different mechanisms and can exert their toxic effects in different ways [5]. It has been a highly debatable subject as to which form of aggregated amyloids exhibits the strongest seeding potency and maximum toxicity. However, an increasingly accepted hypothesis is that oligomeric forms of amyloidogenic proteins such as α-Syn, tau, amyloid-β, and many others are the most toxic intermediates causing impairment in many cellular processes [6–15]. In addition to α-Syn protein pathology, synucleinopathies also exhibit abundant tau pathology in the form of neurofibrillary tangles (NFTs), which has also been long studied in PD [16–18] and dementia with Lewy bodies (DLB) [19, 20]. Tau is a microtubule-binding protein with six alternatively spliced isoforms [21]. Recent studies suggest that intermediate forms of tau aggregation, tau oligomers, are the true toxic species in disease and the targets for therapeutic interventions [22–25]. Our laboratory has extensively studied tau oligomers in several neurodegenerative diseases including Alzheimer’s disease (AD) [11, 12], progressive supranuclear palsy (PSP) [26], traumatic brain injury (TBI) [27], and frontotemporal dementia and parkinsonism linked to chromosome 17 (FTDP-17) [28], as well as in synucleinopathies such as PD and DLB cases [29]. We have previously shown that in addition to the α-Syn oligomers, oligomeric tau is also present in PD and DLB brain tissues. Moreover, we observed that these two oligomeric species coexisted in the same aggregate [29]. In our recent study, we observed that complexes of oligomeric α-Syn and tau isolated from PD brain tissue were more potent, causing behavioral impairment in Htau animals [30]. The presence of overlapping protein pathologies in multiple neurodegenerative diseases, including PD [31, 32], support the phenomenon of protein cross-seeding. Previously, we have demonstrated that α-Syn can cause tau aggregation in vitro [33] and such cross-seeding can result in a more toxic form of tau oligomers [30, 34]. Tau and α-Syn are shown to induce fibrillization of each other in vitro [35].

Amyloidogenic proteins such as amyloid-β and α-Syn can form structurally distinct fibrillar structures in vitro, indicating their polymorphic nature [36, 37]. The occurrence of conformationally distinct amyloid-β deposits has been demonstrated in AD brain tissue by using luminescent conjugated polythiophene probes [38]. Amyloid-β fibrils extracted from the brain tissues of two AD patients showed different structures, indicating the in vivo occurrence of polymorphisms of amyloid-β [39]. Studies have indicated that protein aggregates spread from one brain region to another in a “prion-like” manner [40–42]. The discovery that a single protein such as tau can form different inclusions in different neurodegenerative diseases, collectively known as tauopathies, suggests the presence of “amyloid strains” with distinct properties [40, 43]. To this end, the occurrence of fibrillar tau strains has also been demonstrated in different tauopathies [44]. Also, fibrillar α-Syn isolated from brain tissues of one PD and one multiple system atrophy (MSA) patients showed variable strain characteristics [45].

While the dopaminergic system is important in PD, cholinergic system is also associated with the development of dementia in PD pathogenesis [46, 47]. Different conditions and cofactors have been shown to cause different conformational and aggregation states of α-Syn [48]. Conway et al. showed that the interaction between dopamine (DA) and α-Syn by stabilizing the latter as adducts results in the formation of α-Syn protofibrils [49]. Later, it was demonstrated that in addition to the disaggregation of existing α-Syn fibrils, DA inhibited further fibrilization of α-Syn [50]. Interaction of α-Syn with lipid membranes is a well-known phenomenon. Studies suggest that increased levels of polyunsaturated fatty acids (PUFAs) are associated with oligomerization of α-Syn [51, 52]. Docosahexaenoic acid (DHA), a PUFA, is abundantly present in the brain. It is reported that α-Syn regulates fatty acid metabolism in the brain, and moreover, it binds with cerebral PUFAs, such as DHA [53, 54]. The ability of DHA to form α-Syn oligomers in vitro has also been demonstrated [55, 56].

As most of the amyloid strains’ studies are performed on their fibrillar structures, there is limited knowledge about polymorphic strains of α-Syn and tau in their toxic oligomeric states and the effect of their interaction is still under investigation. Here, we show that α-Syn can form two distinct oligomeric strains by two biological inducers, DA and DHA. To the best of our knowledge, this is the first study where two disease-relevant conditions have been used to establish α-Syn oligomeric strains by thoroughly characterizing and comparing their biochemical, biophysical, and biological properties. Moreover, such unique α-Syn oligomeric strains interact with the tau inducing its distinct aggregations with differential biochemical and biological attributes.

## Methods

### Preparation of α-Syn Oligomers

Recombinant human full-length α-Syn protein was expressed in *E. coli* and purified. Purified protein was dialyzed overnight against water and lyophilized. Lyophilized α-Syn protein was dissolved in 50% acetonitrile as 1 mg/mL and relyophilized. Oligomers of α-Syn without any modification was prepared following our published protocol [29, 33]. Briefly, an aliquot of thus relyophilized protein was dissolved in 280 μl of hexafluoroisopropanol (HFIP) and allowed to incubate at room temperature (RT) for 10–20 min in 2 ml Eppendorf tube. Double-distilled H_2_O was added to this solution to make the final concentration 0.7 μg/μl. The resulting solution was then stirred at 500 RPM with a Teflon-coated micro stir bar for 48 h inside the fume hood at RT closed with a cap with holes to allow the evaporation of HFIP. This oligomeric preparation was used as control α-Syn oligomers (SynO-UM) for comparison purposes.

### Preparation of DA-Modified α-Syn Oligomers

To prepare DA modified oligomers (SynO-DA), we followed a previously published protocol by Lee et al., with modification [57]. Dopamine hydrochloride (Sigma, H8502) was dissolved in 20 mM Tris (pH 7.4) and 100 mM NaCl solution to obtain a final concentration of 100 mM DA solution. Relyophilized α-Syn protein was immediately dissolved in the freshly prepared DA solution at a 1:20 M ratio (protein:DA) to obtain a final protein solution of 50 μM α-Syn:1 mM DA. This solution was incubated at 300 RPM at 37 °C for 110 h. At the end of the incubation, the solution was centrifuged at 16,000 g for 10 min and the supernatant was collected. This fraction was further filtered by using 3 kDa filter unit (Millipore) to remove unbound free DA molecules.

### Preparation of DHA-Modified α-Syn Oligomers

α-Syn oligomers modified by DHA (SynO-DHA) were prepared following published protocol [55]. Briefly, purified and relyophilized α-Syn protein was dissolved in 1× PBS at a concentration of 0.7 μg/μl. Cis-4,7,10,13,16,19-Docosahexaenoic acid (Sigma, 53171) was added to the α-Syn solution at a molar ratio of 1:50 (protein:DHA) and incubated at 37 °C for 48 h at 500 RPM. This fraction was further filtered by using 3 kDa filter unit (Millipore) to remove unbound free DHA molecules.

### Preparation of Fibrils

Fibrils of α-Syn were prepared by following our published method [58]. Briefly, recombinant purified α-Syn was dissolved in water with physiological salt concentration and stirred for 6–7 days at 37 °C. Sodium azide was added at 0.01% to the final solution to avoid bacterial contamination.

### Tau Aggregation Assay

Human recombinant full-length wild-type (WT) 2N4R tau (tau 441) was expressed in *Escherichia coli* and purified as described previously [59, 60]. Tau pellet was denatured with 8 M urea and subjected to overnight dialysis against 1× PBS (pH 7.4). Next, the concentration of tau was measured using the bicinchoninic acid protein assay (Micro BCA Kit, Pierce) and adjusted to 1 mg/mL by adding 1× PBS. Aliquots of 500 μl of tau monomer were made and stored at − 20 °C. Each 500 μl of aliquot (0.5 mg protein) was mixed with 500 μl of 1× PBS. To prepare α-Syn oligomer cross-seeded tau aggregate, seeds of each α-Syn oligomeric strain were added to 1 mL tau monomer (0.5 μg/μl), at 1:140 ratio [33] and incubated for 24 h on an orbital shaker at RT. To prepare unseeded tau oligomers, tau monomer (0.5 μg/μl) was aggregated for 24 h on an orbital shaker at RT.

### Western Blot Analysis

Three different concentrations of α-Syn oligomer preparations, as well fibrillar α-Syn sample were loaded on precast NuPAGE 4-12% Bis-Tris gels (Invitrogen) for SDS-PAGE analysis. For electrophoresis with tau aggregates, approximately 2 μg of each tau aggregate sample was loaded. Gels were subsequently transferred onto nitrocellulose membranes and blocked with 10% nonfat dry milk at 4 °C overnight. Membranes were then probed with primary antibodies, Syn33 (1:4000), LB509 (1:2000; Abcam, Ab27766), T22 (1:250), and Tau 5 (1:5000; BioLegend, 806402) diluted in 5% nonfat dry milk for 1 h at RT. HRP-conjugated, anti-mouse IgG (1:6000, GE Healthcare) was used to detect LB509 and Tau 5 immunoreactivity, whereas an HRP-conjugated anti-rabbit IgG (1:6000, GE Healthcare) was used for Syn33 and T22 immunoreactivity. ECL plus (GE Healthcare) was used to visualize the bands. Densitometric analysis was performed using ImageJ software (National Institutes of Health).

### Size-Exclusion Chromatography

All α-Syn oligomers and aggregated tau preparations were analyzed using the AKTA Explorer system fitted with a Superdex 200 Increase 10/300 GL Column. Degassed deionized water was used as the mobile phase with a flow rate of 0.5 mL/min. Gel filtration standard (Bio-Rad, 51–1901) was used for calibrations. Samples were resolved using absorbance at 280 nm.

### Atomic Force Microscopy

Different oligomer preparations of α-Syn, fibrillar α-Syn, and tau aggregates were analyzed by AFM using a non-contact tapping method with a Multimode 8 AFM machine (Bruker, Billerica MA). Briefly, 3–4 μl of each sample was applied onto a fresh-cleaved mica surface and allowed to adsorb at RT overnight. Mica was then washed with 200 μl of deionized water and air-dried. Images were taken from 5 different areas on the mica surface. AFM images were analyzed by using particles analysis tool of the NanoScope Analysis v1.20rl AFM data processing software to examine the height and diameter of the samples.

### Bis-ANS and Thioflavin T Fluorescence Assays

Three microliters of either α-Syn or tau aggregates (0.5 and 0.6 μg/ μl, respectively) and 247 μl of 10 μM bis-ANS (4,4′-dianilino-1,1′-binaphthyl-5,5′-disulfonic acid, dipotassium salt, Invitrogen) prepared in 100 mM glycine-NaOH buffer (pH 7.4) were added to the wells of 96-well clear-bottomed black plates. Each condition was performed in triplicate. The fluorescence intensity was measured at λ-emission 520 nm upon λ-excitation 380 nm. For Thioflavin T (ThT) assay, 3 μl of protein (0.5 and 0.6 μg/μl, respectively) and 247 μl of 20 μM ThT prepared in 50 mM glycine-NaOH buffer (pH 8.5) were added in triplicates to the wells. Fluorescence intensity was read at λ-emission 490 nm following excitation at 440 nm using a POLARstar OMEGA plate reader (BMG Lab technologies). Each condition for this assay was performed in triplicate.

### Circular Dichroism

Circular dichroism spectra of samples were measured in a spectropolarimeter Jasco-720 (JASCO Inc.) equipped with a temperature controller as published earlier [61]. Spectra were recorded at 0.20-nm intervals with a scan speed of 20 nm/min in a quartz cell of 1 mm pathlength. The protein concentration used was 0.1 μg/μl at RT. Spectra were measured in 1× PBS buffer (pH 7.4) from 195 nm to 250 nm and an average of 3 iterations were recorded for each spectrum. The quartz cell was washed with water and ethanol between every use. Proteins’ secondary structures were estimated from CD spectra using K2D3 software, an updated version of K2D2 software [62].

### Fourier Transform Infrared Spectroscopy

FTIR spectroscopy was performed using NICOLET 6700 FT-IR machine equipped with OMNIC software. Absorption spectrum for each sample was obtained by applying 10 μl of sample between 2 zinc selenium windows secured in a holder. Every sample spectrum was background subtracted. Spectra were recorded at RT. All spectra were corrected for background spectrum of D_2_O. Normalized spectra were plotted from 1500 to 1700 cm^−1^ wavelength with major focus on amide I region from 1600 to 1700 cm^−1^ wavelength.

### Proteolytic Digestion of α-Syn Oligomers by Proteinase K Enzyme

Different oligomer preparations of α-Syn (10–12 μg) were treated with different concentrations of proteinase K enzyme (Sigma) ranging from 1 to 2 μg/mL in the presence of 1× PBS buffer and incubated at 37 °C for 30 min. At the end of incubation time, 1× LDS sample buffer (Invitrogen) was added and heated at 95 °C for 10 min. Samples were immediately transferred onto ice to stop the cleavage reaction followed by loading the digestion products into 4–12% Bis-Tris precast gels (Invitrogen) for SDS-PAGE gel electrophoresis. Samples with all conditions were run in two sets for electrophoresis. Gels with one set of digested samples were processed for silver staining (Pierce Silver Stain Kit, Thermo Scientific; 24,612) to visualize the fragments following manufacturer’s instructions. Another set of digested samples were transferred onto nitrocellulose membrane for Western blot analysis immunolabeled with LB509 antibody to visualize the PK-resistant aggregates.

### Proteolytic Digestion of Aggregated Tau by Proteinase K Enzyme

Aggregated tau samples (~ 3 μg) were treated with proteinase K enzyme at 0–1 μg/mL in the presence of Tris-HCl and NaCl (100 mM and 5 mM final concentrations, respectively) and incubated at 37 °C for 1 h. At the end of incubation time, 1× LDS sample buffer (Invitrogen) was added and heated at 95 °C for 10 min. Samples were immediately transferred onto ice to stop the cleavage reaction followed by loading the digestion products into 4–12% Bis-Tris precast gels (Invitrogen) for SDS-PAGE gel electrophoresis. Western blot analysis with generic tau antibody, Tau 5, was performed to visualize the digested fragments.

### Mass Spectrometry

#### Trypsin Digestion of α-Syn Oligomers

The α-Syn monomer remained in SynO-DA and SynO-DHA preparation was removed by a microcentrifuge filter unit (molecular cutoff 30 kDa) (Millipore). Ten micrograms of SynO-DA and SynO-DHA were added into a filter unit, respectively. Then, 200 μl of 25 mM ammonium bicarbonate (pH 8.0) was added into each filter unit and centrifuged at 12,000×*g* for 10 min. This step was repeated twice. The α-Syn oligomers that remained in the filter were transferred into a 0.6-mL tube and 0.2 μg of trypsin was added into each sample. The sample was incubated at 37 °C for 0.5, 1 and 5 h.

### PRM Analysis of *Rickettsia* Protein RC0497

For parallel reaction monitoring (PRM) analyses, the peptides were analyzed with Easy nLC1000 UHPLC-Q Exactive Orbitrap LC-MS system (Thermo Scientific, San Jose, CA). A 1-h linear gradient from 2% solvent A (0.1% formic acid in water) to 35% solvent B (0.1% formic acid in the acetonitrile) was used for each LC-MS/MS run. The resolution of the full scan was 70,000 (@m/z 200), the target AGC value was set to 3 × 10^6^, and maximum fill time was 200 ms for the full scan; 17,500 (@m/z 200), a target AGC value of 2 × 10^5^, and maximum fill times of 100 ms for MS2 scan. PRM targeted eight tryptic peptides of α-Syn. The assessment of the detection of peptides was performed post-acquisition using Skyline version 3.6.0.9321 [63, 64]. For each peptide evaluated, the signals of the 5–6 most intense fragment ions were extracted from each corresponding MS/MS spectrum. The MS/MS spectra of the fragment ions detected were submitted to spectral matching. The comparison of the relative intensities of these fragments with those defined in the reference composite MS/MS spectrum was performed based on the dot product (dotp) value.

### Primary Cortical Neuron Culture

The C57BL/6 animals (Jackson Laboratory, 000664) were used for primary cortical neuron isolation. Primary cortical neuronal cells from C57BL/6 mice during embryonic days 16–18 were isolated using Accutase solution (Sigma, A6964) and maintained as described elsewhere [65]. Neuronal cells were plated on poly-D-lysine-coated glass coverslips (Corning, Inc.) at a density of 2 × 10^5^ cells/mL in a 24-well plate containing neurobasal medium (Gibco, 12348017) supplemented with 2% B-27, 0.5 mM GlutaMax (Gibco, 35050-061), 10,000 units/mL penicillin, 10,000 μg/mL streptomycin, and 25 μg/mL amphotericin B supplement. Half of the Media changes were performed every 3–5 days by replacing 50% culture media with fresh media. Cells were grown for 10–13 days in vitro (DIV) before experiments.

### Cell Transfection and Treatment with α-Syn Oligomer Strains

EGFP/Puromycin-selective empty plasmid and EGFP/Puromycin-hSNCA (human wild type α-Syn; NM_00146054.1) expression plasmids were designed, generated, and purified by VectorBuilder (Chicago, IL). Human neuroblastoma SH-SY5Y cells were cultured in high glucose Dulbecco’s modified Eagle’s medium (DMEM, Gibco) supplemented 10% fetal bovine serum (Gibco) and 1% penicillin/streptomycin (Gibco). After plating on coverslips, cells were transiently transfected with either EGFP/Puromycin-hSNCA or EGFP/Puromycin-selective empty plasmid DNA using Lipofectamine 2000 (Invitrogen). Briefly, an empirical concentration of plasmids (125 ng) was mixed with Lipofectamine 2000 (2 μl) for 30 min at RT followed by incubation with cells in FBS-deprived DMEM. After 6 h, culture medium was replenished with 5% FBS-supplemented DMEM for 16 h. The next day, cells exposed to different α-Syn oligomeric strains (SynO-DA and SynO-DHA) at a concentration of 0.125 and 0.25 μM. Cells were also treated with a vehicle (empty vector) alone that were used as negative control. Three independent replicate experiments were performed for each experimental condition. Images were captured with a Keyence BZ-800 Microscope and analyzed using BZ-X Analyzer. A Nikon 100X oil immersion objective was used for image acquisition.

### Cell Toxicity Assays

Cell toxicity and cell viability were determined in human neuroblastoma SH-SY5Y cells as well as SH-SY5Y cells overexpressing human wild-type α-Syn, SH-SY5Y^WT-Syn^. Both cell types were cultured and maintained in high glucose Dulbecco’s modified Eagle’s medium (DMEM, Gibco) supplemented 10% fetal bovine serum (Gibco, 16000-044) and 1% penicillin/streptomycin (Gibco). Cytotoxicity was determined by measuring lactate dehydrogenase (LDH) release using Cytotoxicity Detection kit PLUS (Roche, 04744926001) and cell viability was measured by CellTiter 96® Aqueous Non-Radioactive Cell Proliferation Assay (Promega, G5421) following manufacturers’ instructions as previously described. In brief, cells were treated with five different concentrations of α-Syn oligomers: SynO-DA, SynO-DHA, and SynO-UM, as well as fibrillar α-Syn ranging from 0.125 to 1.5 μM and incubated for 16 and 24 h followed by assaying with LDH. Cell viability assay was performed only at 24 h of incubation. For both assays, absorbance was measured at 490 nm with a Polar Star Omega plate reader (BMG Labtech). Each experimental condition was performed in triplicates in three different independent assays. For the MTS assay, the percentage of viable cells was calculated as ((OD_treated_ -OD_un__treated control_)/ OD_untreated control_) × 100. For LDH assay, the percentage of affected cells was calculated following the formula provided by the manufacturer.

Primary cortical neurons grown on 96-well plates were treated with increasing concentrations of the three α-Syn oligomer preparations and α-Syn fibrils (0.05, 0.125, 0.25, 0.5, 1.0, and 1.5 μM) for 16 h. Additionally, we have also treated the neurons with α-Syn monomer for the same time period. The cytotoxicity was measured by evaluating LDH release.

### Primary Neurons Treatment, Immunostaining, and Confocal Microscopy

Primary cortical neurons grown on the coverslips in the 24-well plates were exposed to 0.5 μM α-Syn oligomer strains for 6 h. For the vehicle-treated group, 1× PBS was added to the neuronal cells and incubated for the same time period. After 6 h of incubation, cells were washed 3 times with 1× PBS and fixed with 4% formaldehyde solution for 15 min at RT. Cells were then washed 3 times with 1× PBS followed by permeabilizing with 0.25% Triton X-100 in PBS for 10 min at RT. Cells were blocked in 5% goat serum for 30 min at RT and incubated with primary antibodies, rabbit anti-PSD95 (1:1000; Abcam, Ab18258) and mouse anti-β-III tubulin (1:1000; Abcam, ab78078) at 4 °C overnight. The next day, cells were washed and incubated with secondary antibodies, Alexa fluor anti-rabbit 568 and Alexa fluor anti-mouse 488 (1:1000, Life Technologies) at RT for 1 h. Following 3 washes, coverslips were mounted with ProLong Diamond antifade mounting media with DAPI (Invitrogen). Coverslips with all treatment conditions were imaged under Zeiss LSM 880 confocal microscope using × 63 objective with 405 nm diode laser and argon laser 458/488/514 nm. Z-stacks were built by capturing images from 17 stacks at 0.37–0.41 μm optimal thickness. Each treatment condition was performed in 3 independent experiment and were randomly imaged at five different regions of interest. All images were analyzed by ImageJ (NIH) software.

### Dendritic Spines Analysis

To assess the effects of different α-Syn oligomeric polymorphs on the number of mature synapses, we followed our previously published method [30]. Briefly, primary cortical neuronal cells from embryos of C57BL/6 mice were exposed to vehicle (PBS), SynO-DA, and SynO-DHA. Neuronal cells were then immunostained with PSD95 antibody (Abcam, Ab18258), a post-synaptic density marker protein and βIII-tubulin antibody (Abcam, ab78078), a neuronal marker protein. Five different areas of 20 μm dendritic shafts (without any branches) from each treatment were randomly chosen to count the PSD95 puncta. Images were taken from 5 different cells per treatment group using identical laser power, photomultiplier gain, and pinhole settings for each experiment. Images were analyzed by a researcher who was kept blinded to the experimental conditions. All treatment conditions were imaged under Zeiss LSM 880 confocal microscope using 63x objective with 405 nm diode laser and argon laser 458/488/514 nm. Three independent experimental replicates were performed for each experimental setting. The intensity of mean PSD95 puncta was calculated using ImageJ software (NIH, Bethesda, Maryland, USA). The threshold value for each channel was set same across all experimental conditions. Intensity of PSD95 was determined by subtracting the background. One-way ANOVA followed by Tukey’s post hoc was used to analyze the dendritic spine results.

### Internalization of α-Syn Oligomer Strains

Primary cortical neurons from embryos of C57BL/6 mice were plated in 96-well plates at 4 × 10^4^ cells/mL and exposed to dynasore hydrate (6.5–26 μg/mL; Sigma, D7693) or heparin (50–200 μg/mL; Sigma, H4784) for 30 min. Oligomeric α-Syn strains, SynO-DA and SynO-DHA were added to the cells at 1 μM concentrations and incubated for further 16 h. For each α-Syn oligomeric strain, two different inhibitors were used at three different concentrations [66]. Cytotoxicity was determined by measuring lactate dehydrogenase (LDH) release using Cytotoxicity Detection kit PLUS (Roche, 04744926001).

### Tau RD P301S Biosensor Cell Culture and Seeding Assay

Tau RD P301S biosensor cells (ATCC; CRL-3275) were cultured in DMEM supplemented with 10% FBS, 100 μg/mL penicillin, and 100 μg/mL streptomycin. Cell cultures were maintained in a humidified atmosphere equipped with 5% CO_2_ at 37 °C. To determine the effective dose in seeding assay, a dose-dependent titration experiment was performed in the tau biosensor cells, grown in 96-well plates. After 18 h, cells were transduced with three preparation of aggregated tau with Lipofectamine 2000 (Invitrogen) mixed in Opti-Mem (Gibco) medium following a protocol published elsewhere [67]. Different amounts of each aggregated tau preparation (0.05, 0.125, 0.25, 0.5, and 1.0 μM) were mixed with Liposome and incubated at RT for 30 min before adding to the tau biosensor cells. Cells were incubated for 24 h and 48 h and the fluorescence intensities were measured using a POLARstar OMEGA plate reader (BMG Lab technologies) at the two time points.

For imaging, cells were plated on poly-L-Lysine-coated coverslips at a density of 1 × 10^5^ cells/well in 24-well plates. Cells were exposed to two different concentrations of each tau aggregate strain (0.25 and 0.5 μM) in presence of Lipofectamine 2000 for 24 h followed by washing 3 times with PBS. Coverslips were fixed with 4% formaldehyde and mounted with Prolong Gold mounting media for imaging. Each condition for this assay was performed in triplicate.

### Statistical Analysis

All statistical analyses were performed using Prism 6.0 (GraphPad Software, Inc., San Diego, CA, USA). All values were calculated as mean and standard deviation (SD). Data are presented from at least 3 replicates and from 3 independent experiments. For cytotoxicity assay, average fluorescent intensity measurement and FRET positive cells quantification, two-way analysis of variance (ANOVA) with Bonferroni’s post hoc analysis was performed. For bis-ANS and thioflavin T fluorescence assays, dendritic spine analysis, one-way ANOVA with Tukey’s multiple comparisons test was performed. The number of experiments is mentioned in the figure legends.

## Results

### Characterization of DA- and DHA-Modified α-Syn Oligomers

**Fig. 1:**
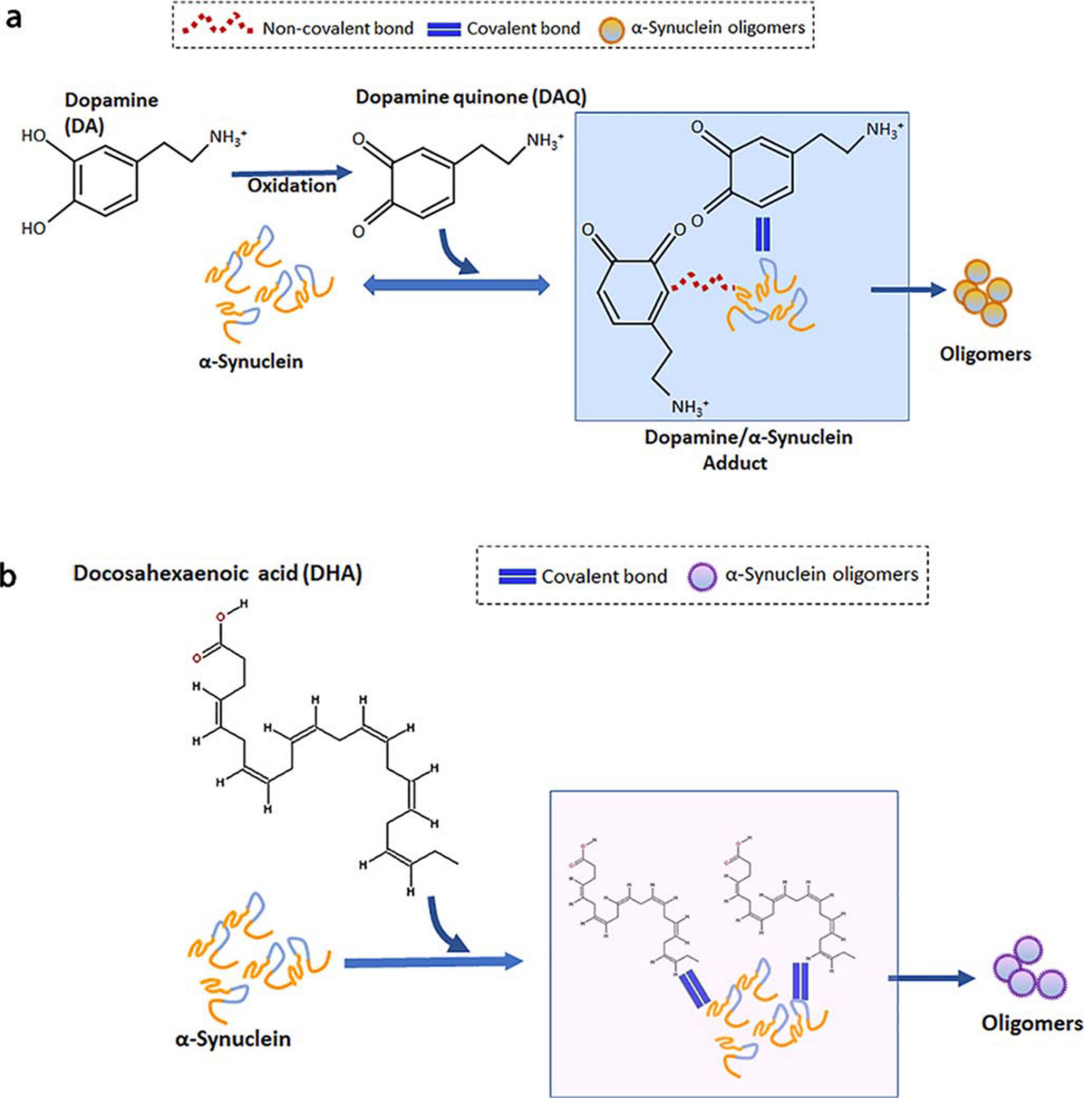
Schematic representation of the two α-Syn oligomeric polymorphs. **a** Generation of α-Syn oligomers by dopamine (DA) modification. Dopamine gets readily oxidized into its quinones, which are then thought to interact with α-Syn by both covalently and non-covalently forming adducts. These adducts result in the formation of modified α-Syn oligomers, hence termed as SynO-DA. **b** Generation of α-Syn oligomers modified by docosahexaenoic acid (DHA), termed as SynO-DHA, primarily via covalent bonding

We have generated oligomers of α-Syn protein by separately modifying the protein with DA and DHA and have thoroughly characterized them to evaluate their polymorphic nature. To generate polymorphic α-Syn oligomeric assemblies, we used purified human recombinant α-Syn protein as previously published [29]. Aggregates of α-Syn were prepared by modifying with DA at 1:20 M ratio of protein to DA following published method by Lee et al. [57, 68]. Upon oxidation, DA forms dopamine quinones (DAQs), which interact with α-Syn forming adducts that finally results in the formation of oligomeric structures (Fig. 1a). The second condition used for α-Syn oligomerization was by modifying the protein with DHA at 1:50 M ratio (protein:DHA). Some populations in DHA-modified α-Syn oligomers were shown to contain covalently bound DHA and oxidative modifications [53, 56] (Fig. 1b). Oligomers of α-Syn formed by modifying with DA and DHA are termed as SynO-DA and SynO-DHA, respectively. For comparison purposes in biochemical analyses, we also prepared α-Syn oligomers without any modification (SynO-UM), following our previously published method [29]. Fibrillar assemblies of human recombinant α-Syn (Syn fibrils) were used to compare all the three oligomeric preparations.

**Fig. 2:**
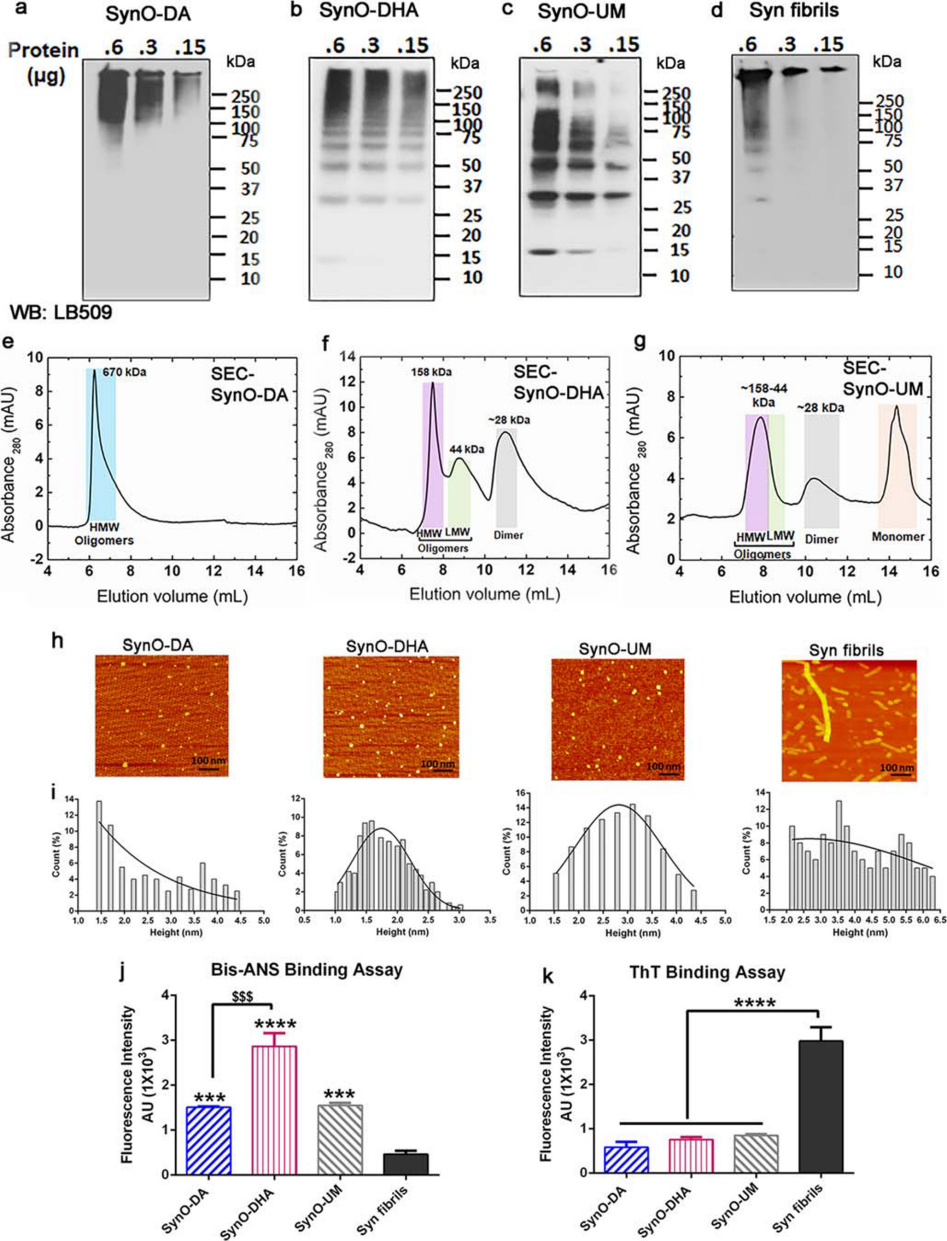
Biochemical characterization of DA- and DHA-modified α-Syn oligomers. **a**–**d** Representative WB images of the indicated amounts of the four different α-Syn aggregates probed with a generic α-Syn antibody LB509. **e**–**g** Size exclusion chromatograms (SEC) of the three oligomers showing different sizes of aggregates. SynO-DA shows more homogeneous aggregates with a single peak, whereas SynO-DHA shows multiple peaks corresponding to different sizes of aggregates. HMW = high molecular weight, LMW = low molecular weight. **h**, **i** Representative AFM images of α-Syn aggregates and their height distribution chromatograms. All three oligomer preparations show spherical structures, whereas Syn fibrils show protofilaments. **j** Fluorescence intensity measurement of bis-ANS binding to α-Syn aggregates shows significantly strong binding to all the three α-Syn oligomers compared to Syn fibrils. SynO-DHA shows strongest binding intensity with bis-ANS than the other two oligomer preparations. **k** Fluorescence intensity measurement of ThT binding to α-Syn aggregates. ThT binding to all three α-Syn oligomer preparations is significantly less compared to Syn fibrils. Data are represented as mean ± SD from three independent experiments. Statistical significance was calculated using one-way ANOVA with Tukey’s multiple comparisons test. ^$$$^*p* < 0.001, ***p < 0.001, ****p < 0.0001. Scale bar 100 nm

SDS-PAGE of SynO-DA, SynO-DHA, SynO-UM, and Syn fibrils, followed by Western blot (WB) analyses with a generic α-Syn antibody, LB509, were performed. We used three different concentrations (0.6, 0.3, and 0.15 μg) of each oligomeric and fibril preparation. WB analysis of SynO-DA showed high molecular weight (HMW) oligomers, mostly ranging from 50 kDa and above (Fig. 2a). This was also evident from the size exclusion chromatography (SEC) of SynO-DA that exhibited a single dominant peak, indicating α-Syn oligomers (Fig. 2e). DHA-modified α-Syn oligomers showed aggregates starting from dimer at ~ 28 kDa to higher than 250 kDa in WB analysis with LB509 antibody (Fig. 2b). Apart from dimers, oligomers formed in this condition were visible as distinctive bands at ~ 50, ~ 70, and ~ 75 kDa that are indicative of different aggregate species. In the SEC chromatogram, SynO-DHA separated into 2 peaks of mostly HMW aggregates, followed by a peak of lower molecular weight (LMW) (Fig. 2f). The SynO-UM sample showed aggregates of different molecular weights in WB analysis with LB509 antibody as well as in SEC (Fig. 2c, g). The WB image of syn fibrils showed a strong band at the top, which is usually noticeable in amyloid fibrils (Fig. 2d). However, it also showed a few weak HMW bands of aggregates. Western blot analyses of all the samples with Syn33 antibody, specific for α-Syn oligomers showed different sizes of aggregates that were consistent with LB509 data (Additional file 1: Fig. S1a-d). Together, the results from the WB and SEC analyses of different preparations of α-Syn oligomers showed that they have different populations of aggregates.

The morphology of the DA- and DHA-modified oligomers was studied by atomic force microscopy (AFM) (Fig. 2h, i). Both the conditions resulted in spherical α-Syn oligomers as shown in the AFM images. The unmodified α-Syn oligomers also showed similar spherical structure, while the fibrillar α-Syn sample mostly revealed protofibrils as well as few long fibrils. The size distribution histograms showed that most of the SynO-DA oligomers had a height of 1.5–2 and ~ 3.75 nm, while the SynO-DHA oligomers had 1.5–2.5 nm (Fig. 2i). The height of SynO-UM and α-Syn fibrils were mostly between 2.5–3.5 and 2–5.5 nm, respectively (Fig. 2i). All the aggregated samples showed differences in their diameter (Additional file 1: Fig. S1e-h). To assess the hydrophobicity and aggregation state of the two oligomeric preparations, we performed fluorescence binding assays of the whole α-Syn aggregate samples using bis-ANS and thioflavin T (ThT). It has been shown that bis-ANS fluorescent dye strongly binds with amyloid oligomers compared to fibrils, while ThT binds strongly with amyloid fibrils rather than with oligomers [29, 69]. In our study, all the three oligomeric preparations showed strong binding with bis-ANS, which was significantly higher than the fibrils (Fig. 2j). However, SynO-DHA showed higher affinity for bis-ANS than SynO-DA and SynO-UM, indicating its increased hydrophobicity. As expected, all three α-Syn oligomers showed low binding affinity for ThT compared to α-Syn fibrils (Fig. 2k). Taken together, the α-Syn oligomers prepared in the presence of DA and DHA showed differences in their biochemical properties.

### DA- and DHA-Modified α-Syn Oligomers Are Structurally Distinct

**Fig. 3:**
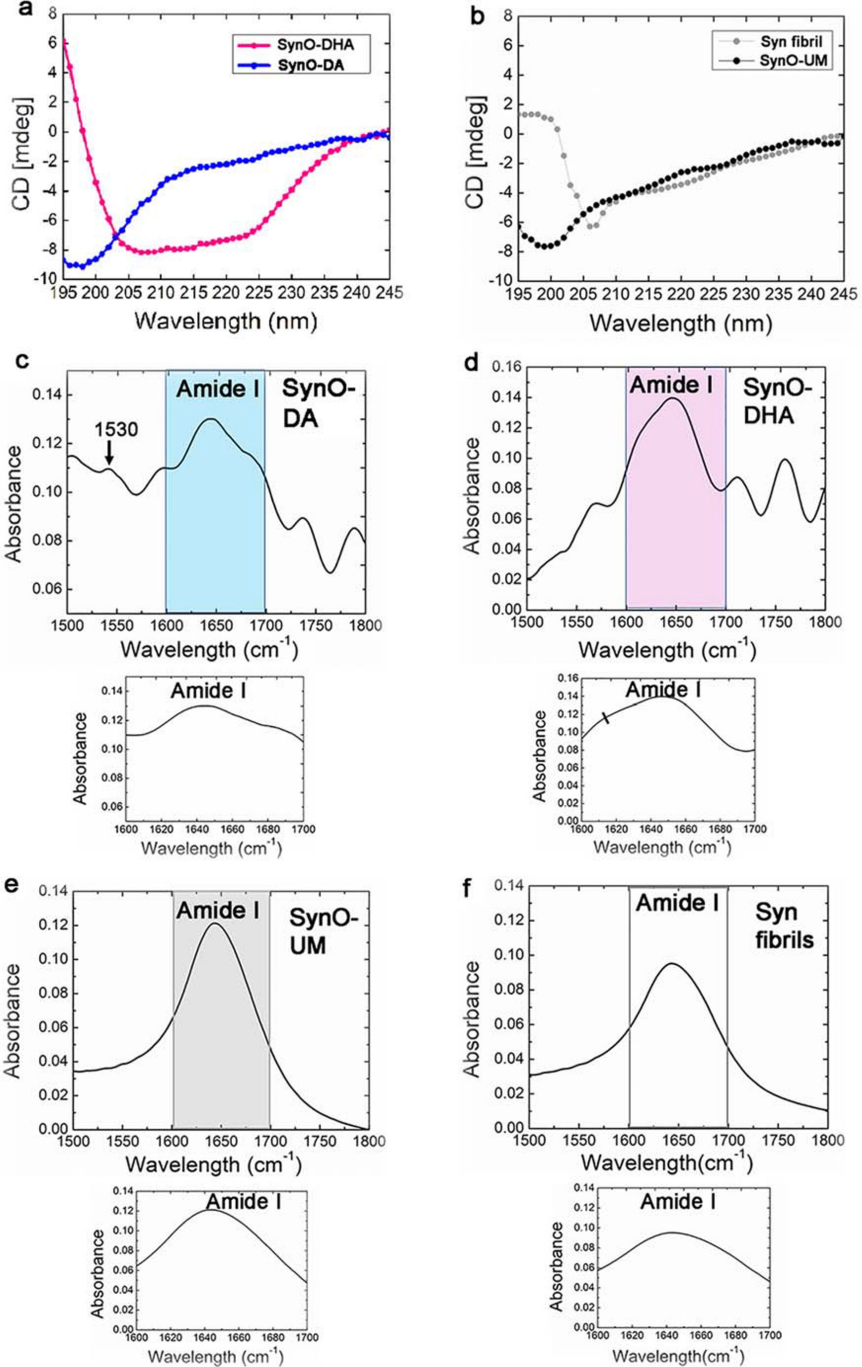
Biophysical characterization of DA- and DHA-modified α-Syn oligomeric polymorphs. **a** CD spectra of SynO-DA and SynO-DHA. SynO-DA shows a minimum around 195 nm indicating mostly random coil, whereas SynO-DHA shows two minima at 208 and 222 nm, suggesting α-helical structure. **b** CD spectra of SynO-UM show random coil and β-sheet, whereas, Syn fibrils showed α-helical and β-sheet structures. **c** FTIR spectrum of SynO-DA with the inset showing 1600 to 1700 cm^−1^ corresponding to the amide I region. This oligomer preparation mostly contains random coil with a peak around 1648–1650 cm^−1^. A small absorption peak at 1530 cm^−1^ in amide II region corresponds to β-sheet structure (marked by black arrow). **d** FTIR spectrum of SynO-DHA shows α-helical structure with an absorption at 1652 cm^−1^. The spectrum also indicates a cross-β-structure (1614 cm^−1^), marked by a black line in the inset. **e**, **f** FTIR absorption spectrum of SynO-UM mostly shows random coil, while an enlarged peak around 1630–1656 cm^−1^ was observed for Syn fibrils, indicative of β-sheet and α-helical structures

To acquire insight into the structural properties of the two oligomeric α-Syn preparations, we determined their secondary structures by spectral analyses using Fourier transform infrared (FTIR) and circular dichroism (CD). The CD spectrum of DA-modified α-Syn oligomers exhibited mostly a random coil structure with a minimum around 195 nm (Fig. 3a). The deconvoluted spectrum showed that in addition to random coil, this preparation also contained ~ 8.6% α-helix and ~ 5.09% β-sheet structures. On the other hand, SynO-DHA showed α-helical structure as its major secondary constituent with two minima around 208 nm and 222 nm (Fig. 3a). The deconvoluted spectrum showed ~ 56.82% α-helix and ~ 26.12% β-sheet structures in this sample. These observations are consistent with previous studies, where DA-modified oligomers mostly contained random coil and DHA-modified oligomers contained α-helix as the main structural components [57, 68, 70]. The CD spectrum of SynO-UM showed a distinct minimum around 195 nm indicating random coils (~ 24.91%) with a maximum around 220 nm, indicative of β-sheet (~ 4.14%) [71] (Fig. 3b). On the other hand, α-Syn fibrils had two minima at approximately 208 nm and in the vicinity of 220 nm and a maximum around 196 nm with ~ 28.84% α-helix content and 13.66% β-sheet structures (Fig. 3b). The CD spectra with a minimum around 218 nm and maximum at 196 nm were also shown for α-Syn aggregates containing β-sheet structures [70]. Our observation here is in accordance with a study, where both WT and mutant α-Syn proteins were shown to form α-helix rich oligomers and protofibrils as intermediary aggregates prior to β-sheet rich mature fibrils [72]. The fibrillar α-Syn preparation used in this study mostly contained protofibrils, as shown in the AFM image (Fig. 2h, i), which supports our observation in the CD analysis. The second derivatives of FTIR spectra for amide I regions of both DA-modified and DHA-modified oligomers showed a major peak. However, spectral region from 1600 to 1700 cm^−1^ (insets) detailed the differences between the secondary structures of these oligomers (Fig. 3c, d). The DA-modified oligomers showed a characteristic peak for random coil structure around 1648–1650 cm^−1^, and a small peak at 1675–1685 cm^−1^, that mostly indicates β-turn [73]. Moreover, SynO-DA showed a small peak at 1530 cm^−1^ in the amide II region, indicating β-sheet structures [74] (Fig. 3c). DHA-modified oligomers displayed a peak at 1652 cm^−1^, resulting mostly from α-helix followed by a deep shoulder at 1695 cm^−1^, representing β-sheet structure (Fig. 3d). Additionally, we noticed a peak at around 1614 cm^−1^, indicating a cross-β structure [56], as detailed in the inset (marked by a black line) of the expanded spectrum from 1600 to 1700 cm^−1^ for amide I region. SynO-UM mostly showed random coils with an absorption spectrum around 1649 cm^−1^ (Fig. 3e), whereas, α-Syn fibrils showed an enlarged peak in the vicinity of 1630–1656 cm^−1^ (Fig. 3f). FTIR spectra around 1631–1635 cm^−1^ and around 1653–1656 have been assigned to β-sheet and α-helical structures of α-Syn aggregates, respectively [72]. Taken together, the results from CD and FTIR spectroscopic analyses indicate that SynO-DA and SynO-DHA are two structurally distinct α-Syn oligomeric polymorphs.

### Oligomeric α-Syn Polymorphs Exhibit Differential Toxicity and Dendritic Spine Pathology

**Fig. 4:**
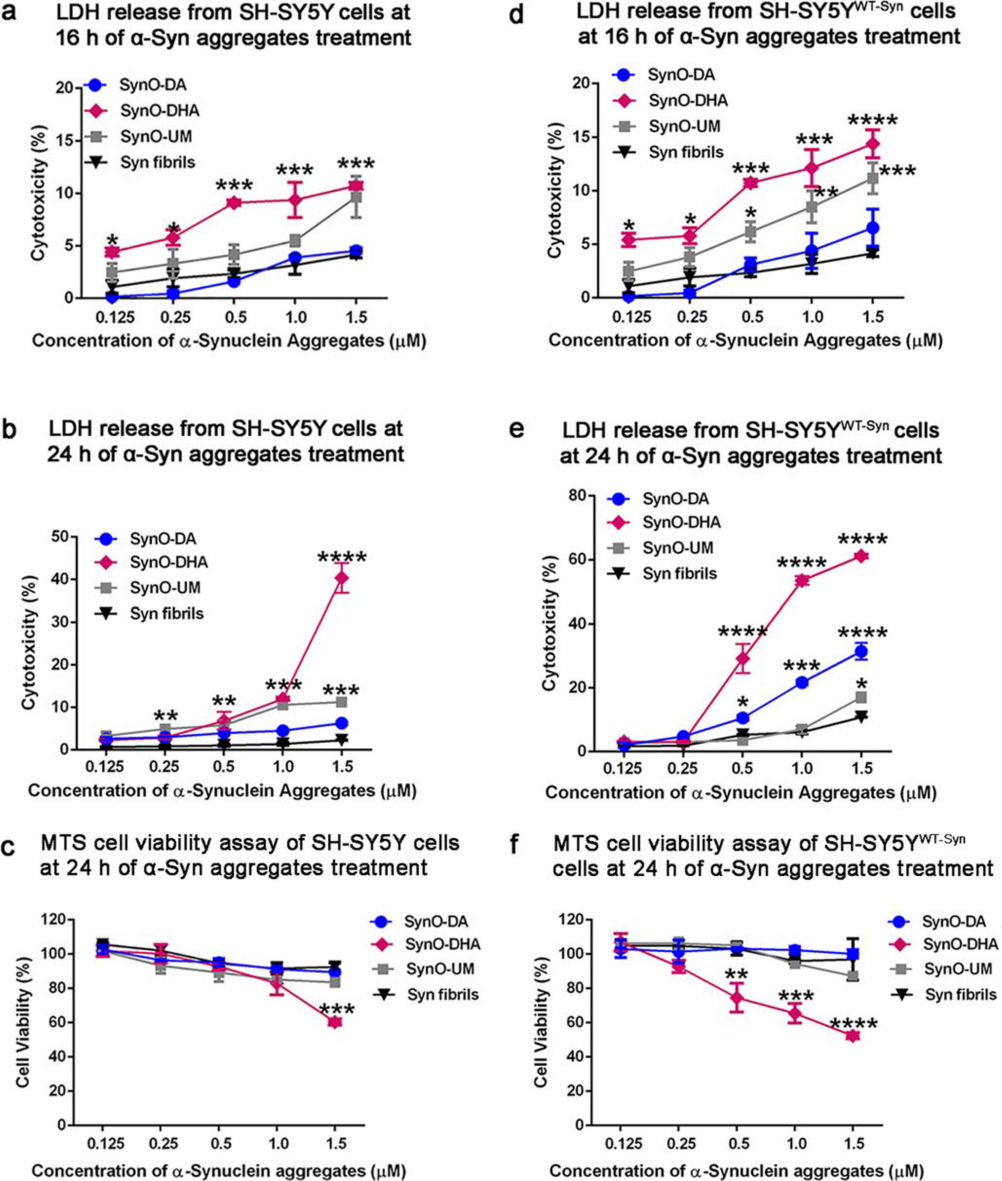
Dose- and time-dependent cytotoxicity induced by α-Syn oligomeric polymorphs. SH-SY5Y cells and the SH-SY5Y^WT-Syn^ cells (SH-SY5Y cells overexpressing human wild-type α-Syn protein) were exposed to 0.125 to 1.5 μM of different α-Syn oligomers preparations for the indicated times. **a**, **b** The effect of different concentrations of α-Syn aggregates on SH-SY5Y cells as measured by LDH release at 16 and 24 h of incubation. **c** Cell viability of SH-SY5Y cells exposed to different concentrations of α-Syn aggregates for 24 h measured by MTS assay. **d**, **e** The effect of α-Syn aggregates on SH-SY5Y^WT-Syn^ incubated for 16 and 24 h as measured by LDH release. **f** Cell viability of SH-SY5Y^WT-Syn^ cells exposed to different concentrations of α-Syn aggregates for 24 h, measured by MTS assay. Data are represented as mean ± SD from three independent experiments. Statistical significance was calculated using two-way ANOVA with Bonferroni post hoc analysis. *p < 0.05, **p < 0.01, ***p < 0.001, ****p < 0.0001

Exogenously added different types of α-Syn oligomers were shown to cause cellular toxicity either by seeding endogenous protein or by acting on cellular membranes, thus elevating intracellular calcium influx [75]. We anticipated that our different oligomer preparations might not possess similar potency to cause cellular toxicity. Therefore, next we sought to assess the dose and time-dependent toxic effects of the two α-Syn oligomers by exogenously adding them to human neuroblastoma cells, SH-SY5Y and the same cell line overexpressing human wild type (WT) α-Syn protein (SH-SY5Y^WT-Syn^). We used five different concentrations of SynO-DA and SynO-DHA as well as SynO-UM ranging from 0.125 to 1.5 μM for 16 h and 24 h followed by measuring lactate dehydrogenase (LDH) release (Fig. 4). To compare the toxicity of the three α-Syn oligomers, α-Syn fibrils were used at the same concentrations and incubated for the same time points. We observed a dose- and time-dependent change in the levels of LDH released in all the oligomers treated groups. In SH-SY5Y cells, SynO-DHA and SynO-UM showed dose-dependent toxicity, which was significantly increased compared to the α-Syn fibrils and was maximum at 24 h of incubation. Although SynO-DA showed a linear dose-dependent increase in LDH release, it did not cause comparable toxicity in these cells at 16 h of incubation. At 24 h of incubation, all the three oligomers showed toxicity compared to α-Syn fibrils (Fig. 4a, b). In SH-SY5Y^WT-Syn^ cells, both SynO-DA and SynO-DHA showed increased dose-dependent toxicity (Fig. 4d, e). Interestingly, SynO-DA was more toxic than SynO-UM in SH-SY5Y^WT-Syn^ cells at 24 h of incubation (Fig. 4e). Additionally, MTS assay was used to estimate the cell viability following 24 h of oligomers treatment, which was reduced in the cells exposed to SynO-DHA, consistent with cytotoxicity assay (Fig. 4c, f). It is noteworthy to mention that, we observed an increased cytotoxicity and a decreased cell viability in the SH-SY5Y^WT-Syn^ cells (Fig. 4e, f) compared to the SH-SY5Y cells (Fig. 4b, c) at 24 h of oligomers treatment. This augmentation in the oligomer-mediated cytotoxicity in SH-SY5Y^WT-Syn^ cells might be driven by the overexpression of the α-Syn protein.

**Fig. 5:**
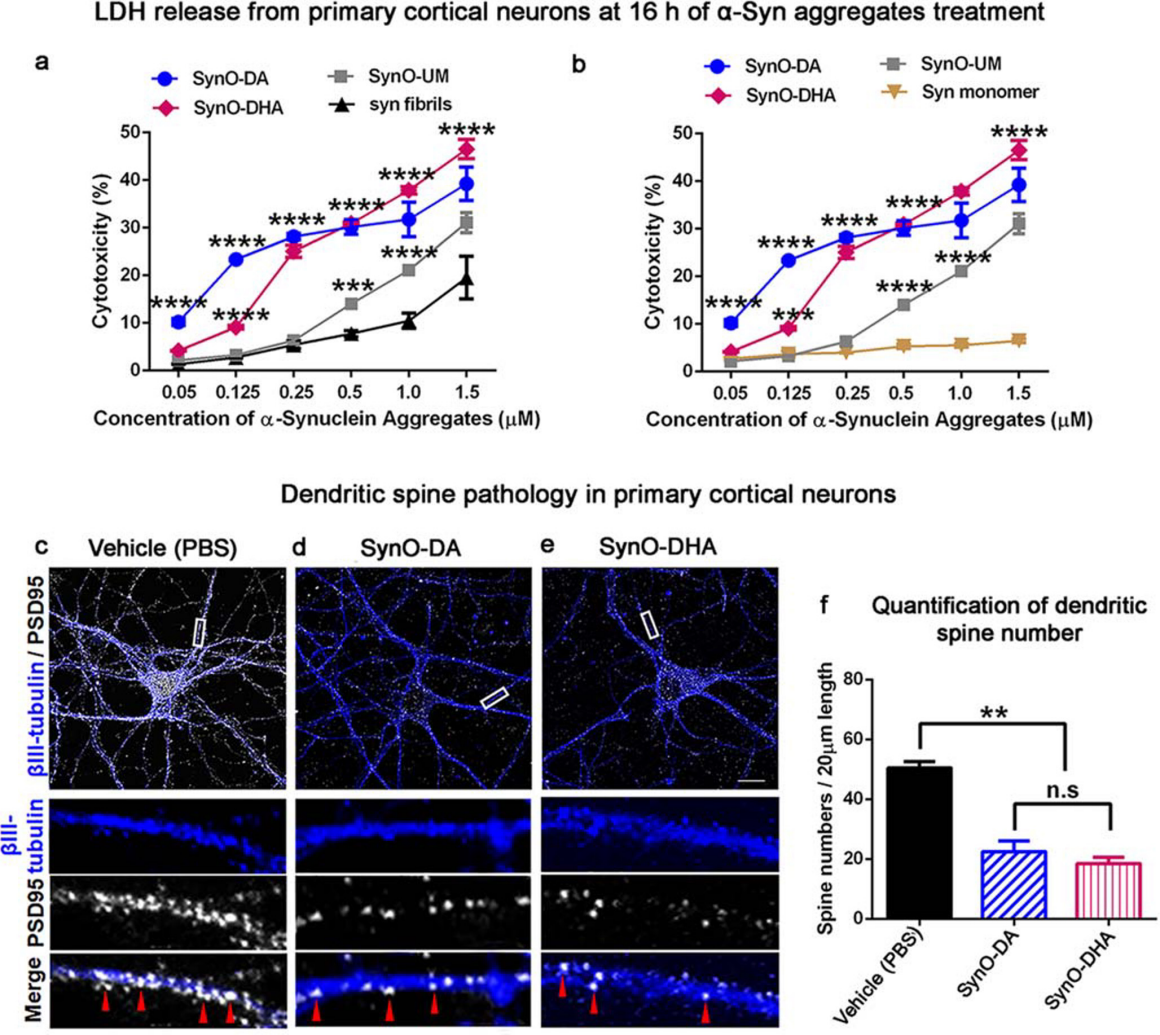
Dose-dependent cytotoxicity and dendritic spine pathology of primary cortical neurons exposed to two α-Syn oligomer polymorphs. **a**, **b** Cytotoxicity in primary cortical neurons exposed to 0.05 to 1.5 μM of different α-Syn oligomers preparations for 16 h was determined by measuring LDH release (in percentage). Neurons exposed to SynO-DA and SynO-DHA showed significant toxicity compared to α-Syn fibrils (**a**) and α-Syn monomer (**b**). SynO-UM also showed significant toxic effects compared to α-Syn fibrils and α-Syn monomer. **c**–**e** Representative confocal microscopic images of primary cortical neurons treated with 0.5 μM SynO-DA and SynO-DHA for 6 h and immunolabeled with marker for postsynaptic density protein, PSD95 (appearing as white puncta) and βIII-tubulin as neuronal marker (blue). The spines are marked by red arrowheads in the merged images. **f** Quantification of PSD95 puncta per 20 μm length of dendritic shaft. Primary neurons treated with the two α-Syn oligomer preparations show significantly decreased number of dendritic spines compared to the vehicle-treated ones. The quantification is represented as mean ± SD from five randomly chosen areas of dendritic shafts from five different cells per treatment group in three independent experiments. Statistical significance was calculated using one-way ANOVA with Tukey’s multiple comparisons test. **p < 0.01. Scale bar 10 μm

The cytotoxic effects of the two α-Syn oligomers were also examined by exogenously adding them to the primary cortical neurons isolated from wild-type C57BL/6 mouse embryos. Primary neurons were exposed to SynO-DA, SynO-DHA, SynO-UM, α-Syn fibrils as well as α-Syn monomer at an increasing concentration (0.05, 0.125, 0.25, 0.5, 1.0, and 1.5 μM) for 16 h (Fig. 5a, b). A dose-dependent increase in the levels of LDH release was noticed with increasing concentration of SynO-DA, SynO-DHA, and SynO-UM compared to the α-Syn fibrils (Fig. 5a) and α-Syn monomer preparations (Fig. 5b). To further assess the functional roles of the two α-Syn oligomeric polymorphs, primary cortical neurons grown on coverslips were exposed to vehicle (PBS), and the two α-Syn oligomeric polymorphs for 6 h. Cells from all groups were immunostained with antibody for postsynaptic density protein 95 (PSD95) and βIII-tubulin antibody for neurons, followed by imaging with confocal microscopy (Fig. 5c–e). Both the oligomeric polymorphs reduced the number of dendritic spines, visualized as puncta of PSD95 positive structures. The reduction in the dendritic spines was significant in both SynO-DA and SynO-DHA treated groups compared to the vehicle treatment (Fig. 5f). Taken together, these data suggest that DA- and DHA-modified α-Syn oligomers have distinct cellular consequences.

### DA- and DHA-Modified α-Syn Oligomeric Polymorphs Reveal Distinct Sensitivity to Proteinase K

**Fig. 6:**
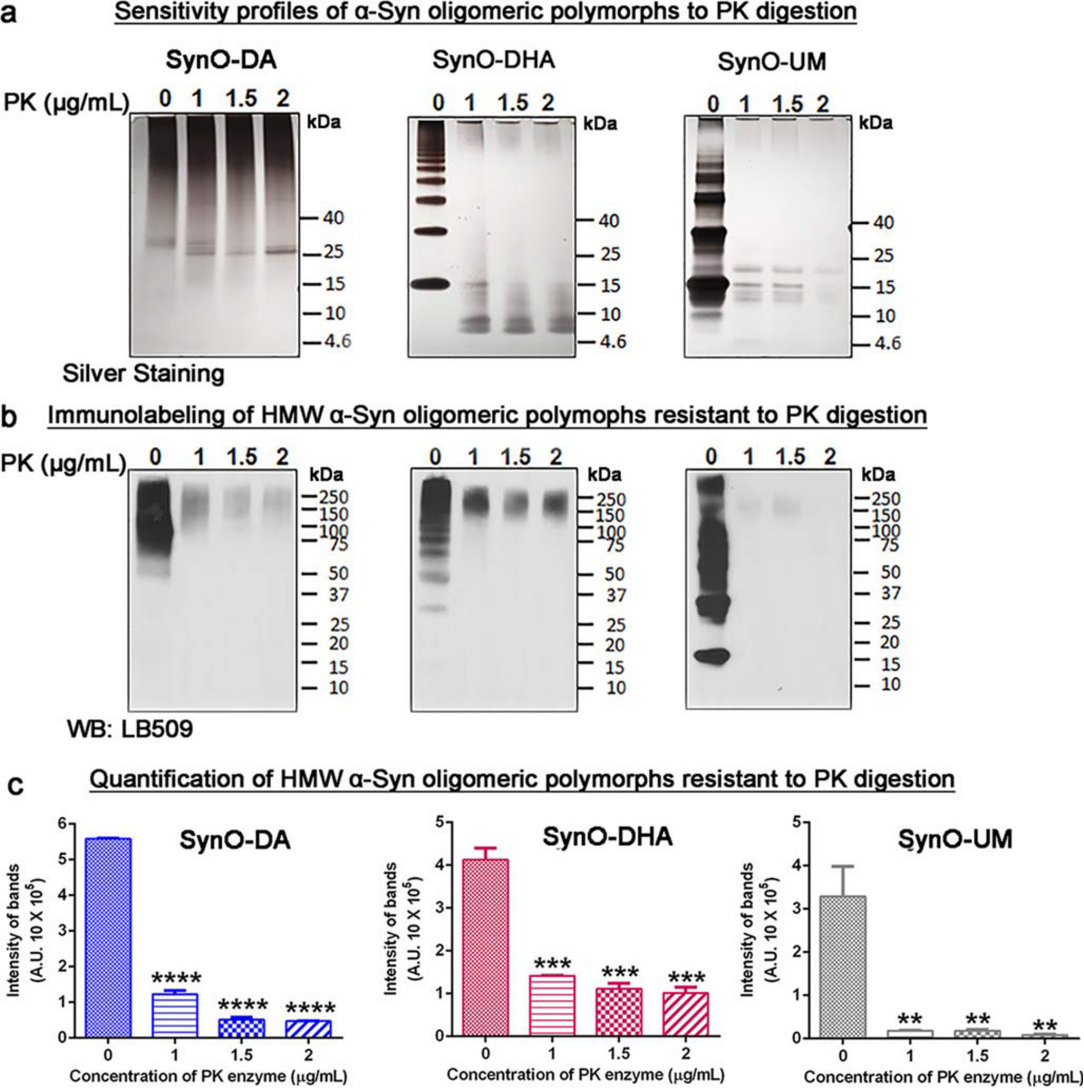
Proteolytic digestion profiles of α-Syn oligomeric polymorphs. **a** Silver staining images of α-Syn oligomers, SynO-DA, SynO-DHA, and SynO-UM digested with 1, 1.5, and 2 μg/mL proteinase K (PK) enzyme. **b** Representative WB images of the same set of samples as in **a**, immunolabeled with LB509 antibody, showing the PK-resistant high molecular weight (HMW) aggregates. **c** Densitometric quantification of the undigested bands (HMW aggregates) of **b**. Undigested bands from each sample were compared with the band from untreated sample (0 PK). The histograms represent mean ± SD from three independent experiments. Statistical significance is calculated using one-way ANOVA with Tukey’s multiple comparisons test. **p < 0.01, ***p < 0.001, ****p < 0.0001

In the previous sections, we have established that the two oligomeric polymorphs are different in their aggregate size, hydrophobicity and biological properties. Furthermore, to evaluate the conformational differences between the two oligomeric polymorphs as well as their stability as oligomers, we measured their sensitivity for proteinase K (PK) enzyme digestion. PK digestion had long been used in classifying strains of prion fibrils [76, 77]. Nevertheless, this method has been extended and widely used for identifying other amyloid strains, such as amyloid-β, α-Syn, and tau fibrils [78]. We treated SynO-DA, SynO-DHA, and SynO-UM with increasing concentrations of PK enzyme (0–2 μg/mL). All the digested samples were then run in SDS-PAGE followed by silver staining. The pattern of fragments generated by PK digestion provides information on the stability of the oligomers, as well as its core. We observed that SynO-DA was resistant to PK, thus indicating a stable core of these oligomers (Fig. 6a). By contrast, SynO-DHA was sensitive to PK showing a fragmentation pattern that was different from the SynO-UM preparation. To show the nature of HMW aggregates following PK digestion, we simultaneously performed WB analysis with the same set of PK-digested samples probed with LB509 antibody (Fig. 6b). The HMW bands that were more resistant to PK in the three oligomer samples, were detected based on their epitope availability for LB509 antibody. These HMW aggregates were quantified and compared with the undigested counterparts for all the three samples (0 PK) (Fig. 6c). The PK-resistant HMW aggregates of SynO-DA were also visible in WB analysis. SynO-DHA showed partially undigested HMW aggregates that decreased with increased concentration of PK enzyme, indicative of differences in the HMW aggregates in the SynO-DHA and SynO-DA samples. The signal for HMW aggregates in SynO-UM was less strong in silver staining and in WB analyses. These data together indicate that SynO-DA and SynO-DHA are the two distinct strains of α-Syn oligomers.

Furthermore, to support our observation from PK digestion, we performed mass spectrometry (MS) analysis of trypsin digestion of SynO-DA and SynO-DHA. The two α-Syn oligomer strains were digested with trypsin for 0.5, 1, and 5 h under native condition. The amino acid sequence of human α-Syn protein is shown in Additional file 2: Fig. S2a. The trypsin cleaves peptides on the C-terminal side of lysine and arginine amino acid residues. We analyzed eight tryptic peptides generated from SynO-DA and SynO-DHA with parallel reaction monitoring (PRM)-MS (Additional file 2: Fig. S2b). The sequence coverage of the MS analysis is 74% including peptides from N-terminal, C-terminal and the middle region of the α-Syn. The MS intensity of each peptide originated from SynO-DA and SynO-DHA is shown in the Additional file 2: Fig. S2c-j. The abundance of the tryptic peptides of SynO-DA and SynO-DHA varied depending on the location of the peptide in the sequence of α-Syn and the types of inducer used for α-Syn oligomerization. The MS intensities of the T13–23 (EGVVAAAEKTK) N-terminal peptide originated from SynO-DA and SynO-DHA were almost the same (Additional file 2: Fig. S2c), suggesting that the trypsin-accessibility to the N-terminal lysine residues on the two oligomers are very similar. On the contrary, the trypsin-accessibility to the C-terminal lysine residues on SynO-DA and SynO-DHA appeared to be significantly different. For example, after 0.5 h of digestion with trypsin, SynO-DA produced about 64 times more C-terminal peptide T97–140 than SynO-DHA (Additional file 2: Fig. S2h), implying that the conformation of SynO-DA may hinder the accessibility of Lys92 to trypsin. We observed the similar phenomenon for C-terminal peptide T98–140. The lysine residues in the middle of α-Syn also displayed some differences in trypsin-accessibility (Additional file 2: Fig. S2d-g). These results suggest that the conformational differences between SynO-DA and SynO-DHA differentially affect the proteolysis of the oligomers.

### α-Syn Oligomeric Strains Show Different Seeding Potencies of Cytosolic α-Syn Protein

**Fig. 7:**
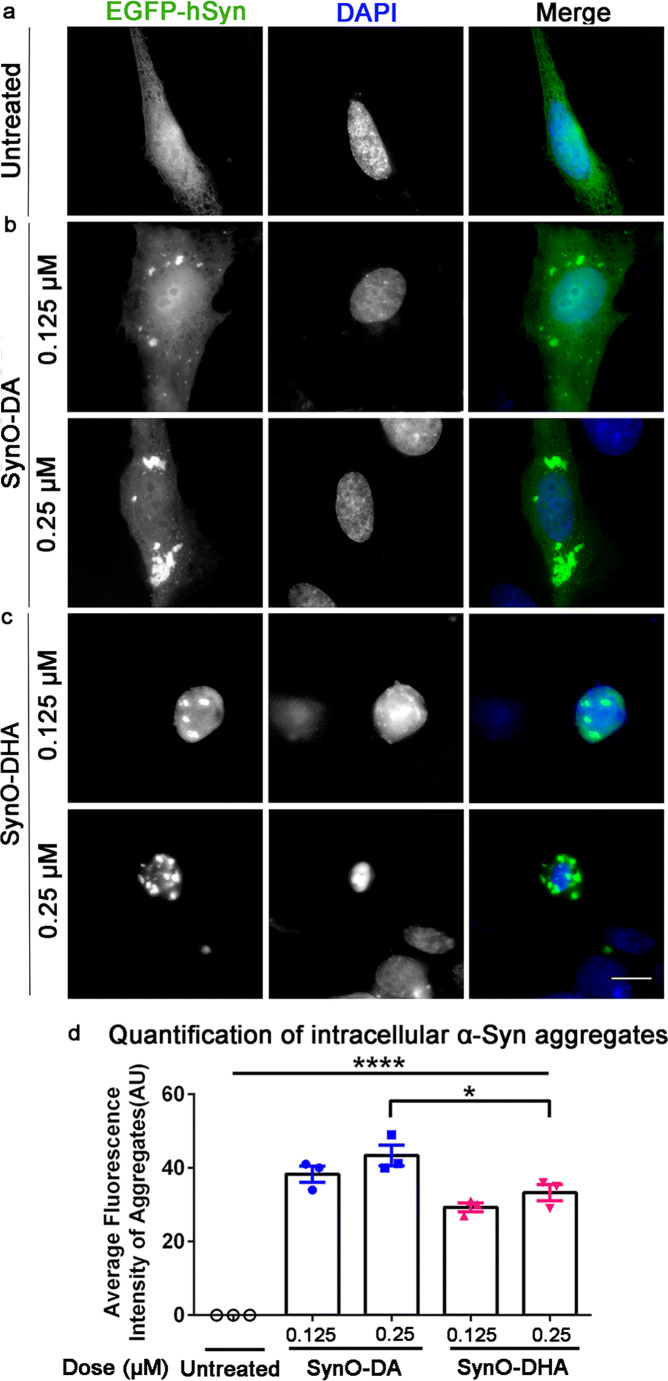
Seeding potency of α-Syn oligomeric strains. **a**–**c** Representative epifluorescence microscopic images of transiently EGFP-hSyn expressing SH-SY5Y cells exposed to SynO-DA and SynO-DHA at 0.125 and 0.25 μM concentrations for 16 h. EGFP-hSyn (green) and DAPI (blue; nuclei) are shown in gray. The merged images on right panels show cytosolic α-Syn aggregates formed by seeding with the different concentrations of α-Syn oligomeric strains: SynO-DA (**b**) and SynO-DHA (**c**). Cells expressing EGFP-hSyn but not exposed to α-Syn oligomers do not show formation of aggregates. **d** Quantification of average fluorescence intensity of α-Syn aggregates calculated from fifteen different regions of interest (ROIs) in three different fields from five independent experiments. The histograms represent mean ± SD. Statistical significance is calculated using two-way ANOVA with Bonferroni post hoc analysis. *p < 0.05, ****p < 0.0001. Scale bar 10 μm

One of the key phenomena in amyloid strains is that the strains act as seeds in the recipient cells, thus recruiting endogenous protein into the aggregation and augmenting the degeneration of cells. Therefore, to investigate whether the two α-Syn oligomeric strains have seeding potency, we used SH-SY5Y cells transiently transfected to express human wild-type α-Syn protein linked to enhanced green fluorescence protein (EGFP-hSyn), SH-SY5Y^EGFP-hSyn^. Two different concentrations of SynO-DA and SynO-DHA (0.125 and 0.25 μM) were exogenously added to the SH-SY5Y^EGFP-hSyn^ cells as seeds and incubated for 16 h. The dose of the oligomers and the incubation time point were chosen based on the toxicity results shown in Fig. 4. Strikingly, both SynO-DA and SynO-DHA were able to recruit cytosolic EGFP-hSyn protein into aggregates (Fig. 7b, c; Additional file 3: Fig. S3). Aggregates were observed as bright green deposits at both the two concentrations used (Fig. 7d). No aggregation was seen in untreated cells (Fig. 7a).

### Dynamin and HSPG Antagonists Inhibit Internalization of α-Syn Oligomeric Strains and Reduce Oligomer-Induced Cytotoxicity in Primary Neurons

We have demonstrated that the two α-Syn oligomeric strains act as seeds for cytosolic α-Syn protein aggregation. Next, we sought to investigate whether the two oligomeric strains can be internalized into the cells via same or different mechanisms. We exposed primary cortical neurons to pharmacological inhibitors for dynamin-dependent (dynasore) and heparan sulfate proteoglycans (HSPGs)-mediated (heparin) endocytic pathways. Cortical neurons were incubated for 30 min in presence of three concentrations of each inhibitor as well as in the absence of any inhibitor. After the incubation time, cells were exposed to 1 μM α-Syn oligomeric strains and incubated further for a total of 16 h. Toxicity induced by the α-Syn oligomeric strains in presence and absence of inhibitors was assessed by detecting LDH release from the cell culture media (Additional file 4: Fig. S4). Both dynasore and heparin significantly blocked the internalization of the oligomeric strains, thereby, rescuing the cells from oligomer-induced toxicity. We observed less toxicity from SynO-DA with increasing concentration of the two inhibitors, indicating that DA-modified oligomers were internalized via dynamin- and/or HSPGs-mediated endocytosis (Additional file 4: Fig. S4a**)**. Similarly, the toxicity from SynO-DHA was significantly rescued in presence of both Dynasore and Heparin inhibitors (Additional file 4: Fig. S4c). Representative bright field images of the primary neurons treated with SynO-DA and SynO-DHA in the absence and presence of dynamin inhibitor are shown in Additional file 4: Fig. S4b, d. We observed that the morphological alterations in primary neurons caused by the toxic effect of the oligomers were rescued when the cells were pre-treated with dynamin inhibitor. These results suggest that, as the internalization of the α-Syn oligomeric strains are inhibited by the pharmacological inhibitors, oligomer-associated cytotoxicity is prevented.

### α-Syn Oligomeric Strains Cross-seed into Different Tau Aggregate Strains

**Fig. 8:**
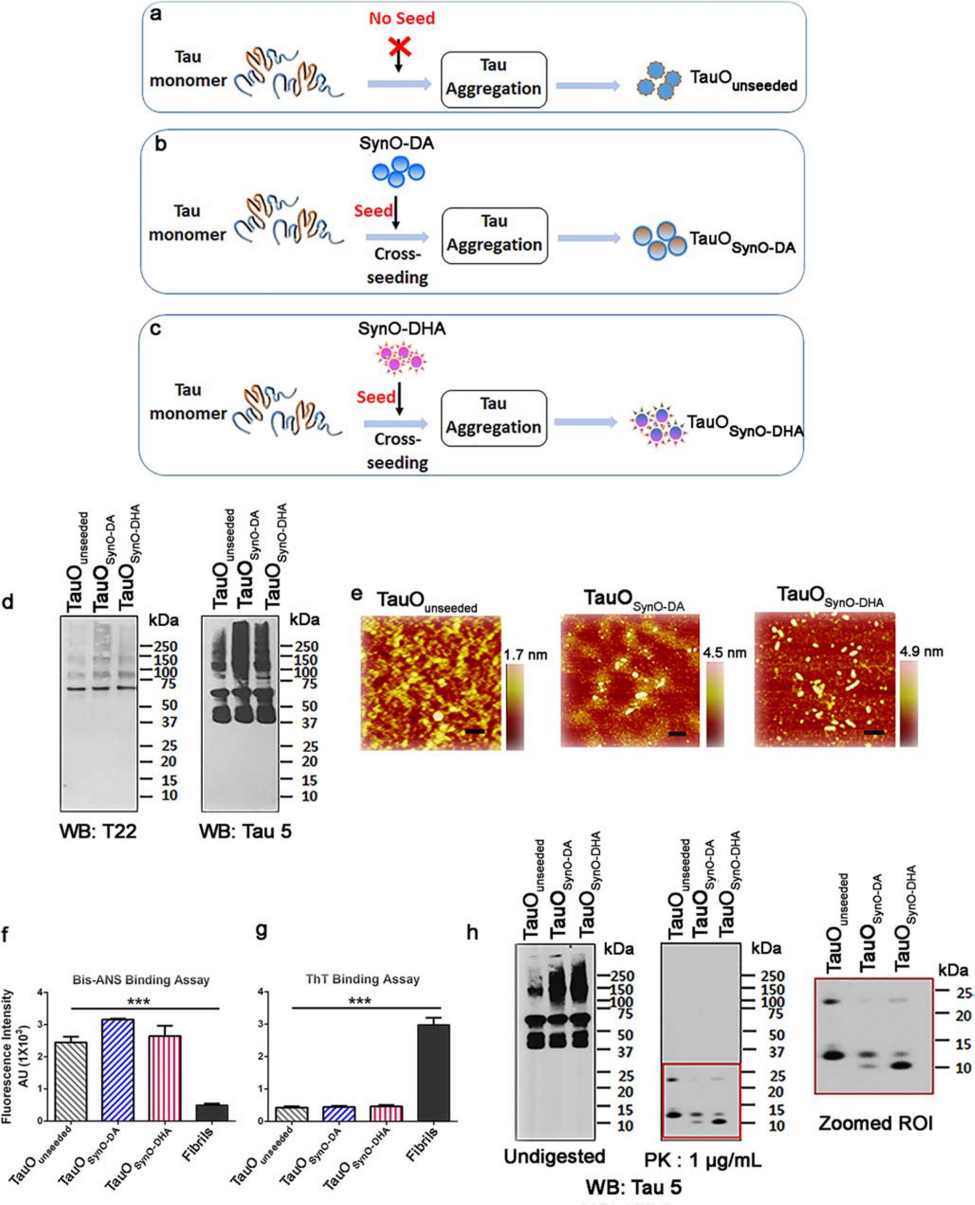
Biochemical analyses of tau aggregates formed by cross-seeding with SynO-DA or SynO-DHA oligomer strains. **a**–**c** Schematic representation of tau aggregates formed without any seed as well as cross-seeded with α-Syn oligomeric strains, SynO-DA and SynO-DHA. The tau aggregates are referred as TauO_unseeded_, TauO_SynO-DA_, and TauO_SynO-DHA_, respectively. **d** Representative WB image of the tau aggregates with tau oligomer specific antibody T22 showing higher molecular weight aggregates. Sequence-specific tau antibody Tau 5 detects different forms of tau aggregates in these samples. **e** AFM images of unseeded and cross-seeded tau aggregates. **f** Fluorescence intensity measurement of bis-ANS binding to all tau aggregates. Bis-ANS binding to all three preparations of tau aggregates is significantly strong compared to fibrils. **g** Fluorescence intensity measurement of ThT binding to tau aggregates. Unseeded and the two cross-seeded tau aggregates show less binding affinity for ThT compared to fibrils. **h** PK digestion profile of the tau aggregates in the WB with Tau 5 antibody showing different cleavage patterns. Zoomed region of interest (ROI) shows fragments generated from three aggregated tau samples after PK digestion. Data are represented as mean ± SD from four replicates performed in three independent experiments. Statistical significance was calculated using one-way ANOVA with Tukey’s multiple comparisons test. ***p < 0.001. Scale bar 100 nm

Previously, we have demonstrated that the α-Syn oligomers act as seeds initiating tau aggregation by forming tau oligomers. Such α-Syn oligomer cross-seeded tau oligomers appeared to be toxic on different cell lines, including primary cortical neurons [30]. Therefore, next we sought to investigate the roles of the two α-Syn oligomeric strains in tau aggregation process. We used strains of α-Syn oligomers as seeds to initiate aggregation of monomeric tau which are referred to as TauO_SynO-DA_ and TauO_SynO-DHA_ based on their respective seeds as schematically illustrated in Fig. 8b, c. As a control, we also aggregated monomeric tau without any α-Syn oligomer seed which is referred to as TauO_unseeded_ (Fig. 8a). Representative WB image of the three tau aggregates probed with T22 antibody showed aggregates of molecular weight of ~75 kDa and above (Fig. 8d). However, TauO_SynO-DA_ showed stronger signal appearing above 150 kDa than TauO_SynO-DHA_ or TauO_unseeded_. Upon probing with Tau 5, a generic sequence specific anti-tau antibody, both TauO_SynO-DA_ and TauO_SynO-DHA_ showed strong signal around ~150 kDa and higher molecular weight compared to TauO_unseeded_. Nevertheless, Tau 5 antibody signal was consistent with T22 for TauO_SynO-DA_ which was higher than TauO_SynO-DHA_.

To evaluate if there were any morphological differences between the two cross-seeded tau aggregates, we performed AFM analysis. Representative AFM images showed that TauO_SynO-DA_ and TauO_SynO-DHA_ contained aggregates higher than TauO_unseeded_ (Fig. 8e). These aggregates were also analyzed by SEC (Additional file 5: Fig. S5a-c). Although, the patterns of the peaks corresponding to the aggregates appeared to be similar for the two cross-seeded tau aggregates, but the elution time was different. Binding of bis-ANS to the three tau aggregates was significantly higher compared to the fibrils (Fig. 8f). Notably, TauO_SynO-DA_ showed stronger binding affinity for bis-ANS than TauO_SynO-DHA_. As expected, ThT bound strongly to the fibrils compared to the three oligomeric aggregates (Fig. 8g). We also determined the secondary structures of the tau aggregates by FTIR spectroscopy (Additional file 5: Fig. S5d-f).

Finally, we evaluated the sensitivity to proteolysis of the tau aggregates by PK digestion following our published method [79]. Tau aggregates were digested with PK enzyme at 1 μg/mL concentration followed by WB analysis with Tau 5 antibody (Fig. 8h). Intriguingly, the two cross-seeded tau aggregates, TauO_SynO-DA_ and TauO_SynO-DHA_ showed completely different patterns of fragments upon PK digestion, indicating their differences in the protease-sensitive cores. Thus, the cross-seeded tau aggregates varied in their stability and conformation, suggesting them as two different strains of tau aggregates.

### Cross-seeded Aggregated Tau Strains Exhibit Distinct Tau Seeding

**Fig. 9:**
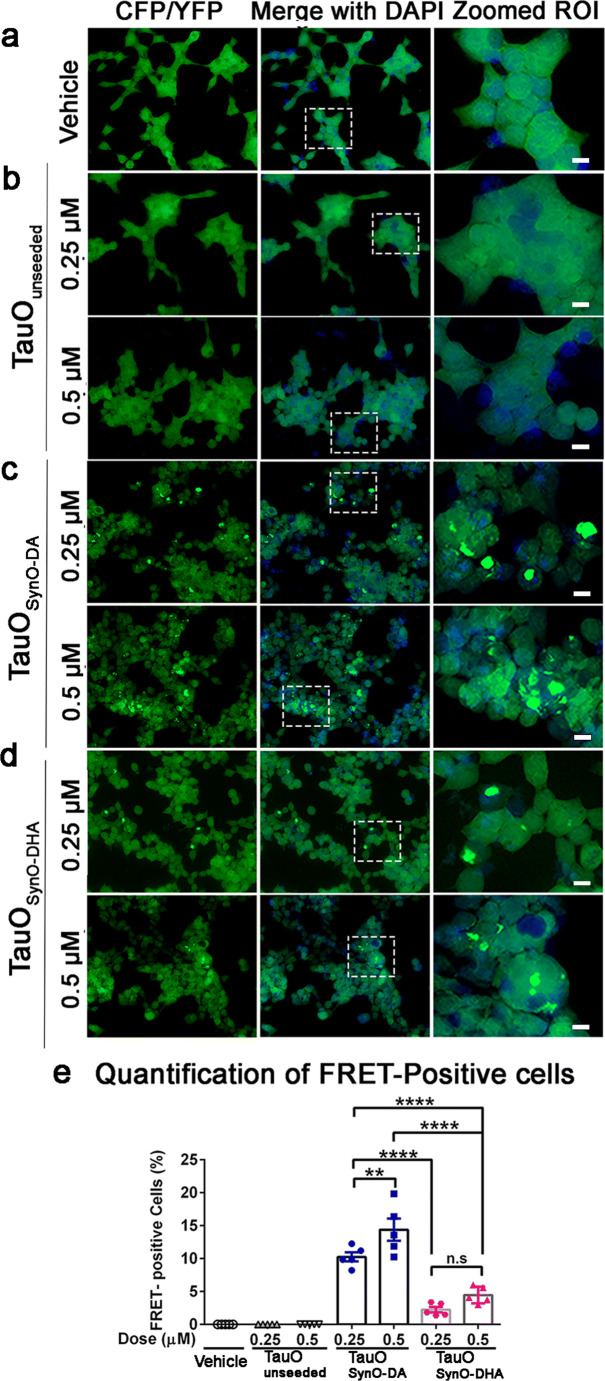
Seeding assay of tau aggregate strains in Tau RD-P301S CFP/YFP FRET biosensor cells. **a**–**d** Tau biosensor cells were exposed to the cross-seeded tau aggregates or unseeded tau aggregate at 0.25 and 0.5 μM concentrations for 24 h with Lipofectamine. The cross-seeded tau aggregates, TauO_SynO-DA_ and TauO_SynO-DHA_ show dose-dependent increased seeding propensity at the concentrations used resulting in the formation of cytosolic tau aggregates. Unseeded tau aggregates do not show any seeding. TauO_SynO-DA_ has more potent seeding efficiency than TauO_SynO-DHA_. **e** Quantification of FRET positive cells observed in all 4 groups of treatment: vehicle, TauO_unseeded_, TauO_SynO-DA_, and TauO_SynO-DHA_. Quantification is performed from fifteen regions of interest (ROIs) from three replicates performed in three independent experiments. The histograms represent mean ± SD. Statistical significance is measured by using two-way ANOVA with Bonferroni post hoc analysis. **p < 0.01, ****p < 0.0001. Scale bar 10 μm

Previously, it has been shown that the tau aggregates present in human and mice brain lysates contained the effective seed causing tau aggregation in the Tau-RD P301S-CFP/YFP FRET biosensor cells [67, 80]. Next, we sought to investigate whether the two aggregated tau strains generated here can act as seeds for tau aggregation. The effective dose for seeding activity of the aggregated tau strains (TauO_SynO-DA_ and TauO_SynO-DHA_) was empirically determined by generating dose-response curves (Additional file 6: Fig. S6). Tau biosensor cells were exposed to increased concentrations (0.05, 0.125, 0.25, 0.5 and 1 μM) of the three tau aggregates in the presence of Lipofectamine and the fluorescence intensity was measured at 24 h and 48 h time points. For both the cross-seeded tau aggregates, fluorescence intensity was detected at 0.25, 0.5, and 1 μM concentrations at 24 h (Additional file 6: Fig. S6a) and 48 h (Additional file 6: Fig. S6b), indicative of the tau inclusion formation. It is noteworthy to mention that at 48 h time point, even 0.125 μM concentration of seed was able to produce significantly increased fluorescence intensity compared to 0.05 μM concentration of seed. On the contrary, TauO_unseeded_ did not produce any detectable fluorescence at either 24 or 48 h. Based on this observation, we exogenously added TauO_SynO-DA_ and TauO_SynO-DHA_ at 0.25 and 0.5 μM concentrations for 24 h to the tau biosensor cells grown on coverslips (Fig. 9a–d). Simultaneously, the unseeded tau oligomers were also used at the similar concentrations. We observed that the two cross-seeded tau aggregates acted as seeds forming tau inclusions in the biosensor cells at both the concentrations, while TauO_unseeded_ did not. Since apparently, there was no tau aggregates formed in the vehicle or TauO_unseeded_ treated cells, we compared the seeding between TauO_SynO-DA_ and TauO_SynO-DHA_ treated groups. Although at lower concentration of 0.25 μM, these tau aggregates were able to seed, the seeding was increased at 0.5 μM concentration (Fig. 9e). More notably, TauO_SynO-DA_ was more effective seed than TauO_SynO-DHA_, showing significantly increased seeding capacity at 0.25 μM concentration than TauO_SynO-DHA_ at 0.5 μM concentration. As mentioned above, TauO_unseeded_ did not generate any visible seeding at the concentrations and the incubation time tested here, implying that longer incubation time might be required. These results suggest that the tau aggregate strains possess different seeding capacities.

## Discussion

To date, several laboratories have demonstrated the polymorphic nature of the fibrillar amyloid β and α-Syn as well as tau fibrils. However, polymorphism of α-Syn and tau in their toxic oligomeric conformation is still under investigation. In this study, we report the formation of two biologically relevant α-Syn oligomeric strains modified by physiological inducers such as DA and DHA. The occurrence of the oligomeric α-Syn and tau co-aggregates in PD and DLB brain tissues [29] point to the cross-seeding phenomenon. Therefore, here we have also demonstrated the effects of the distinct α-Syn oligomeric strains in tau aggregation. To the best of our knowledge, this is the first study demonstrating such distinct biologically relevant α-Syn oligomeric strains with differential consequences in tau aggregation by cross-seeding, thus resulting in discrete tau aggregate strains.

The reasons behind selective vulnerability of dopaminergic neurons being affected in PD pathology are not clearly known. However, one of the proposed mechanisms points to the possible modification of α-Syn by DA [81]. Studies have also reported a substantial loss of cholinergic neurons in the different brain regions and also serotonergic neurons of the raphe nuclei in PD pathogenesis [82]. Furthermore, hippocampal cholinergic projections were shown to contain Lewy pathology in DLB [83], and this pathology was also associated with the cognitive decline in PD with dementia [84]. Hence, the possibility of an equally important role of non-dopaminergic neurons in PD and DLB pathologies has been highlighted in several studies. A well-known characteristic of α-Syn protein is its ability to interact with lipid membranes. It has been shown that α-Syn can form oligomers by interacting with DHA, an abundantly expressed PUFA in the brain [85]. Here, we have demonstrated that the effects of DA and DHA modification on α-Syn oligomerization can lead to the polymorphism of α-Syn oligomers with differential biological consequences. Biochemical analyses of the two α-Syn oligomeric preparations revealed the differences in their aggregate size and hydrophobicity. The toxicity of the oligomers has been shown to correlate with their size as well as their increased surface hydrophobicity measured by the bis-ANS binding assay [86]. Previous study has shown that treating human α-Syn protein expressing SH-SY5Y cells with polyunsaturated fatty acids can induce the formation of SDS-stable α-Syn oligomers causing cytotoxicity [51]. In our study, DHA-modified α-Syn oligomers showed significant dose- and time-dependent increase in cytotoxicity compared to the DA-modified oligomers. A possible explanation could be that the surface hydrophobicity of DHA-modified oligomers is higher than that of the DA-modified oligomers, which was observed from the bis-ANS fluorescence binding assay, despite their similar spherical morphology as shown by AFM. From spectroscopic analyses, we observed that the two α-Syn oligomeric polymorphs have different secondary structures: DA modified oligomers mostly contain random coil, while DHA modified oligomers have α-helix as the main component. This observation is consistent with previous findings [57, 68, 70]. The full-length α-Syn has been shown to adopt β-sheet and cross β-sheet structures in oligomers and mature fibrils, respectively [9, 87]. Upon its interaction with lipid membranes, α-Syn adopts α-helical structures [88], and it has been demonstrated that α-Syn oligomers acquire α-helical structures as an intermediate state of aggregation prior to mature fibril formation [72, 89]. These helix-rich oligomers exhibited more cytotoxic effects than the compact β-sheet rich α-Syn fibrils [72]. Consistent with this observation, α-helix rich SynO-DHA was more toxic to the SH-SY5Y, SH-SY5Y^WT-Syn^ cells and primary cortical neurons, compared to the SynO-DA or SynO-UM in our study. Moreover, both SynO-DA and SynO-DHA showed significant reduction of dendritic spines in mouse primary cortical neurons. This observation supports the toxic effects of the oligomers since dendritic spine pathology is one of the most commonly occurring events in neurodegenerative diseases [90, 91].

Digestion of the α-Syn oligomers with PK enzyme reveals the differences in their sensitivity to proteolysis. DA-modified oligomers show resistance to proteolysis, while DHA-modified oligomers are sensitive showing cleaved fragments, suggesting that these two oligomeric polymorphs can be considered as strains. Additionally, tryptic digestion of the two oligomers followed by mass spectrometry analysis show different cleavage patterns in the two oligomer preparations, further suggesting differences in their stability. Interestingly, both DA- and DHA-modified oligomers can seed cytosolic α-Syn protein into aggregates at different levels. However, further studies will be required to clearly understand the seeding effects of the two strains. Our observation of cytosolic protein seeding is in agreement with a previous study where DA-modified α-Syn oligomers seeded cytoplasmic α-Syn in a reporter neuroblastoma N2A cell line [92]. There is no study showing such seeding effect of DHA modified oligomers. It has been shown that extracellular α-Syn oligomers impair the lysosomal degradation pathway, leading to its intracellular accumulation [93]. Here we did not study the clearance mechanism of the two α-Syn oligomeric strains. Therefore, further studies in this aspect may provide more insight into the understanding of the toxicity mediated by DA- and DHA-modified α-Syn oligomers. Taken together, our biochemical and biophysical analyses suggest that DA- and DHA-modified α-Syn oligomers are the two distinct strains with different conformation, stability and biological functionalities.

Recent studies have shown that cell-to-cell spreading of the pathogenic protein aggregates is necessary for propagation of the diseases [94, 95]. Heparan sulfate proteoglycans (HSPGs), a family of proteins containing one or more sulfated glycosaminoglycan (GAG) heparan sulfate (HS) have been shown to play an important role in cellular uptake of the aggregated proteins, such as fibrils of amyloid-β [96] and tau [97]. Both fibrillary α-Syn and exosome-associated oligomeric α-Syn were shown to be internalized in cells via HSPGs [67, 98]. A recent study suggests that α-Syn oligomers can also be internalized in human neuroglioma cell line H4 via clathrin-mediated endocytic pathway [93]. Primary cortical neurons were shown to uptake oligomeric forms of α-Syn via dynamin-dependent clathrin-mediated endocytic pathway [99]. Defects in clathrin-mediated endocytic pathway is considered as a susceptibility factor in PD and parkinsonism [100]. In our study, since both the α-Syn oligomeric strains were able to successfully seed cytosolic α-Syn protein aggregation, we were interested in examining the internalization mechanisms favored by these oligomers. We observed that the toxicity induced by the oligomeric strains was rescued in the presence of pharmacological inhibitors such as Dynamin and Heparin for dynamin-dependent and HSPGs-mediated pathways, respectively. This suggests that both strains were internalized via dynamin-dependent and/or HSPGs-mediated endocytosis. However, there was variability in the degrees of dynamin-dependent and HSPGs-mediated endocytosis of the two oligomeric strains. Further investigation is required in this aspect to elucidate the intracellular fate of these oligomeric strains after being internalized.

Pathogenic protein aggregates can act as seeds inducing toxic accumulation of the same protein or other aggregation-prone proteins, thus representing an overlap between multiple protein pathologies. In this study, we have also explored the effect of α-Syn oligomeric strains in tau aggregation. Tau aggregates cross-seeded with the two α-Syn oligomeric strains exhibit differences in their biochemical and biophysical properties. Remarkably, they showed different patterns of fragmentation upon digestion with PK, suggesting their variability in conformation and stability. The Tau-RD P301S-CFP/YFP biosensor cell line developed by Diamond et al. provides a useful tool to measure the seeding activity as a functional aspect of tau aggregates [80, 101]. In our study, we have investigated the seeding propensity of the two aggregated tau strains by exogenously adding them to the tau biosensor cells. We anticipated that not every α-Syn oligomer strain would lead to a biologically relevant tau aggregate strain. Surprisingly, our findings here demonstrate that tau cross-seeded with DA-modified α-Syn oligomers is a more potent seed causing increased tau aggregation. Although, DA-modified α-Syn oligomeric strain is less toxic in cultured cell lines than DHA modified α-Syn strain, the tau aggregate cross-seeded with DA-modified α-Syn oligomer strain is a biologically more effective seed.

## Conclusion

Both DA and DHA are two extremely relevant biological inducers associated with PD pathogenesis. Overall, our findings provide useful insights into the functional crosstalk between the oligomers of α-Syn and tau aided by the biological conditions that might have pathological significance. Our study further suggests that DA modified α-Syn oligomers can lead to a distinct tau aggregate formation and such interaction can lead to increased toxic effects in PD pathogenesis. Our findings regarding the strain-specific interaction between α-Syn and tau would open new avenues for neuroprotective intervention strategies for PD by specifically targeting these stable toxic oligomers.

## Electronic supplementary material

Fig. S1 Biochemical and biophysical characterization of α-Syn aggregates. (**a-d**) WB analyses of α-Syn aggregates probed with Syn33 antibody, an α-Syn oligomer specific antibody. (**e-h**) AFM histograms of α-Syn aggregates showing their diameter. Scale bar 100 nm. (PNG 728 kb)

High Resolution Image (TIF 835 kb)

Fig. S2 Mass spectrometry (MS) analysis of trypsin digested fragments of the two α-Syn oligomeric polymorphs. SynO-DA and SynO-DHA were digested with trypsin for 0.5, 1 and 5 h under native condition. The resulting tryptic peptides were analyzed by mass spectrometry. The intensity of each peptide was normalized with the proteotypic peptide of α-Syn, EGVLYBGSK. (**a**) The amino acid sequence of α-Syn. (**b**) Tryptic peptides of SynO-DA and SynO-DHA that were analyzed by LC-MS. The relative intensities of tryptic peptides are shown: T13-23 (**c**), T33-43 (**d**), T44-58 (**e**), T46-58 (**f**), T81-96 (**g**), T97-140 (**h**), T98-140 (**i**), and T103-140 (**j**). (PNG 530 kb)

High Resolution Image (TIF 432 kb)

Fig. S3 Seeding potency of α-Syn oligomeric strains. (**a-c**) Representative brightfield and epifluorescence microscopic images of transiently EGFP-hSyn expressing SH-SY5Y cells exposed to SynO-DA and SynO-DHA at 0.125 and 0.25 μM concentrations for 16 h. Brightfield images merged with EGFP-hSyn (green) and DAPI (blue; nuclei) are shown on the left panels. Merged immunofluorescence images on right panels showed cytosolic α-Syn aggregates formed by the seeding with the different concentrations of α-Syn oligomeric strains: SynO-DA (**b**) and SynO-DHA (**c**). Scale bar 10 μm. (PNG 1446 kb)

High Resolution Image (TIF 1566 kb)

Fig. S4 HSPG and dynamin antagonists reduce α-Syn oligomeric strains internalization and cytotoxicity in neurons. Primary cortical neurons were pre-treated with three different concentrations of the two inhibitors: Dynasore (6.5-26 μg/mL) or Heparin (50-200 μg/mL) for 30 min. α-Syn oligomeric strains, SynO-DA and SynO-DHA were exogenously added to the cells at 1 μM concentrations and further incubated for a total of 16 h. (**a, c**) Cytotoxicity induced by SynO-DA (**a**) and SynO-DHA (**c**) in absence and presence of the two inhibitors was assessed by measuring LDH release. Internalization of oligomers was blocked in presence of both the inhibitors, thus rescuing oligomers induced toxicity. (**b, d**) Representative live cell images of the primary cortical neurons exposed to SynO-DA (**b**) and SynO-DHA (**d**) in presence and absence of the Dynasore inhibitor. Oligomer-induced toxicity was rescued when cells were treated in presence of Dynasore inhibitor. The quantification is represented as mean ± SD from three independent experiments. Statistical significance was calculated using one-way ANOVA with Tukey’s multiple comparison test, **** p<0.0001. Scale bar 10 μm. (PNG 502 kb)

High Resolution Image (TIF 532 kb)

Fig. S5 Characterization of cross-seeded and unseeded tau aggregates. **(a-c**) Size exclusion chromatograms (SEC) showing peaks of different sizes of tau aggregates. (**d-f**) FTIR absorption spectra of all three tau aggregates with insets detailing the amide I region. (PNG 428 kb)

High Resolution Image (TIF 486 kb)

Fig. S6 Dose-response curves for seeding activity of tau aggregates. Tau biosensor cells were exposed to increased concentrations of the three tau aggregates (0.05, 0.125, 0.25, 0.5 and 1 μM) in presence of Lipofectamine and fluorescence intensity was measured at 24 h (**a**) and 48 h (**b**) time points. Data are represented as mean ± SD from four experimental replicates. Statistical significance was calculated using two-way ANOVA with Bonferroni post hoc analysis. ** p<0.01, *** p< 0.001, **** p<0.0001. (PNG 211 kb)

High Resolution Image (TIF 223 kb)
