# Supplementary figures and images for: Polymorphic α-Synuclein Strains Modified by Dopamine and Docosahexaenoic Acid Interact Differentially with Tau Protein
Source: Mol Neurobiol. 2020 Apr 29;57(6):2741–65. doi: 10.1007/s12035-020-01913-6 (PMC7253398; doi:10.1007/s12035-020-01913-6)

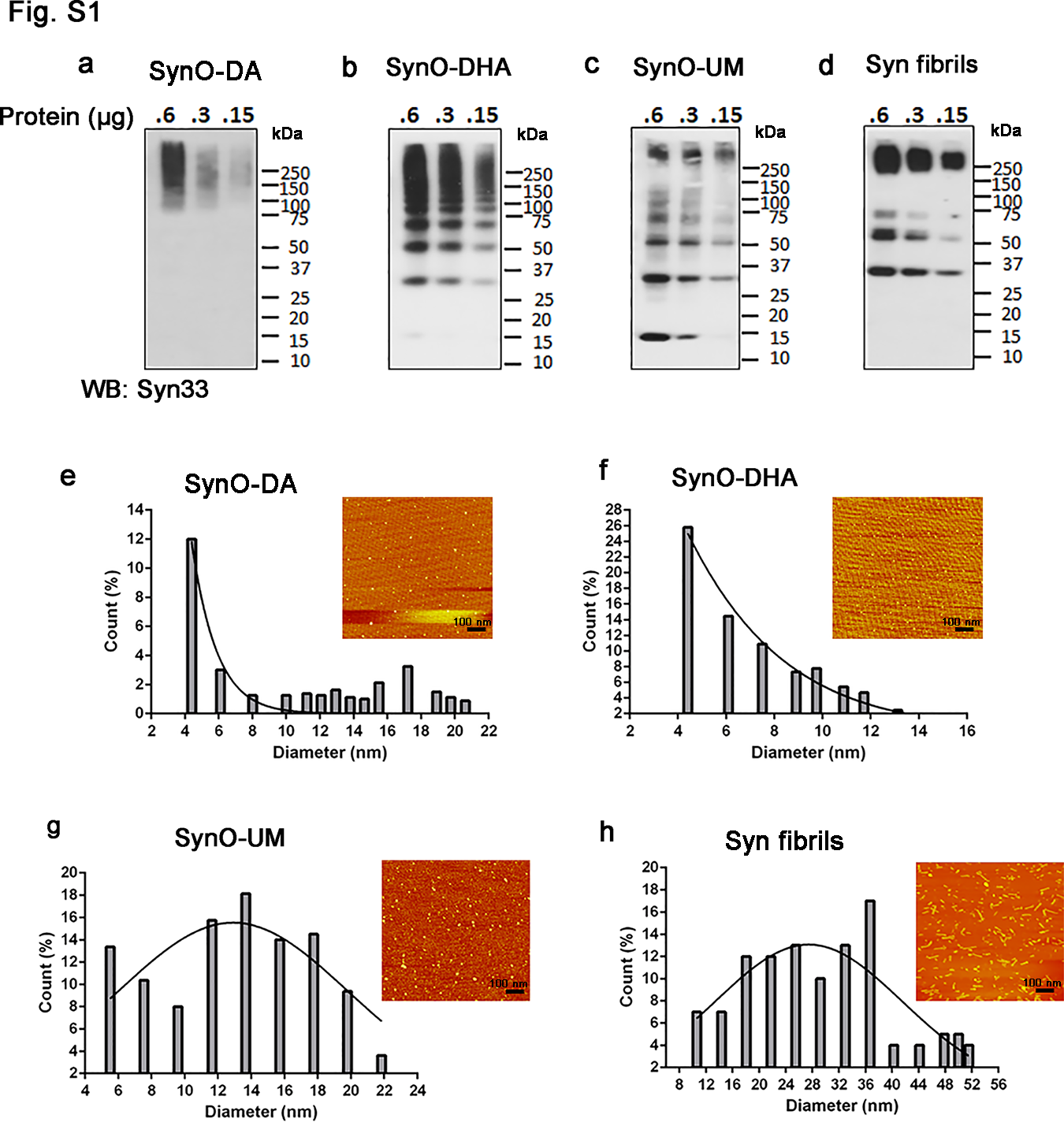

Supplement: Supplementary file 1 — Biochemical and biophysical characterization of α-Syn aggregates. (a-d) WB analyses of α-Syn aggregates probed with Syn33 antibody, an α-Syn oligomer specific antibody. (e-h) AFM histograms of α-Syn aggregates showing their diameter. Scale bar 100 nm. (PNG 728 kb) [file 12035_2020_1913_Fig10_ESM.png]

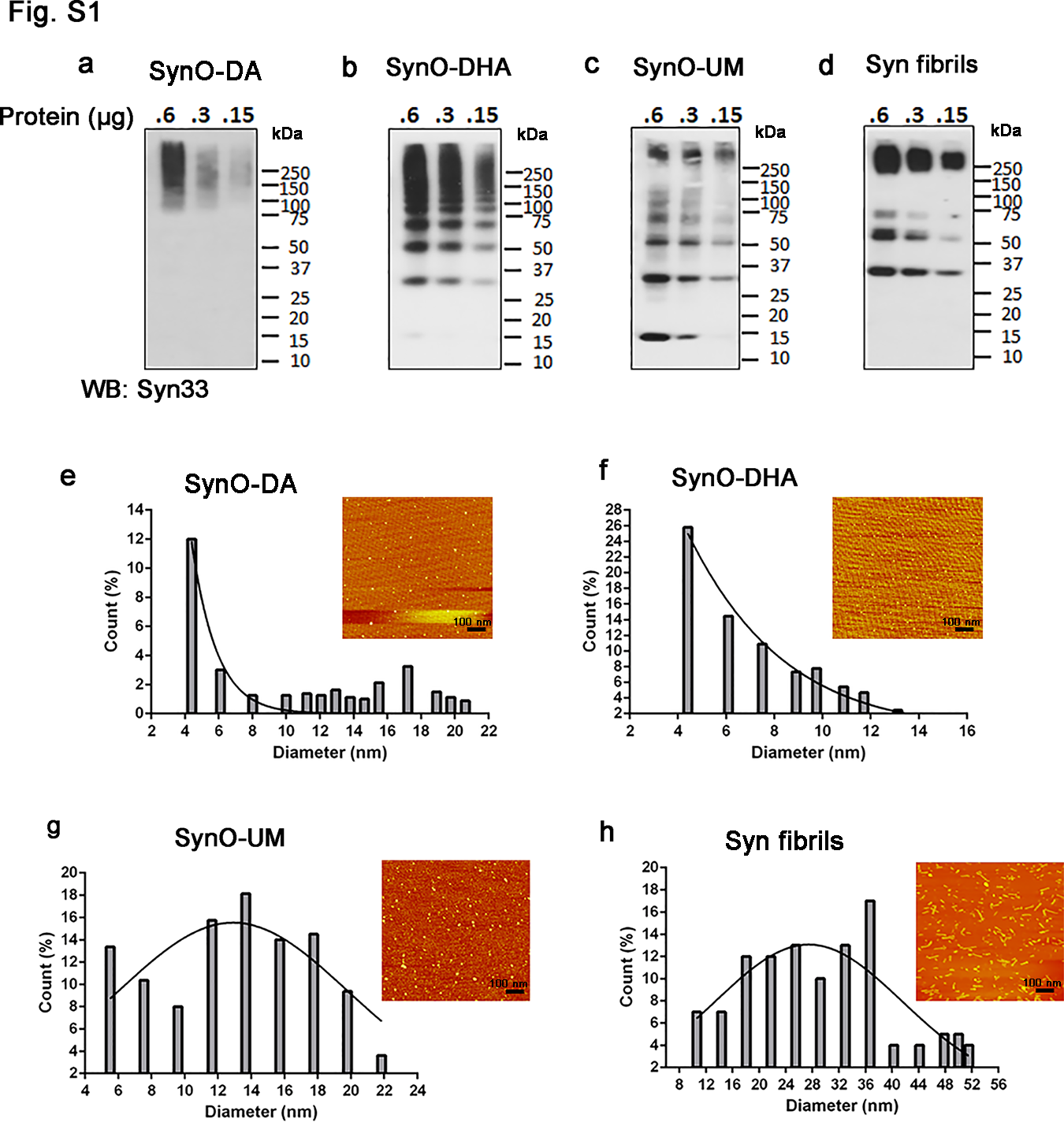

Supplement: Supplementary file 2 — High Resolution Image (TIF 835 kb) [file 12035_2020_1913_MOESM1_ESM.tif]

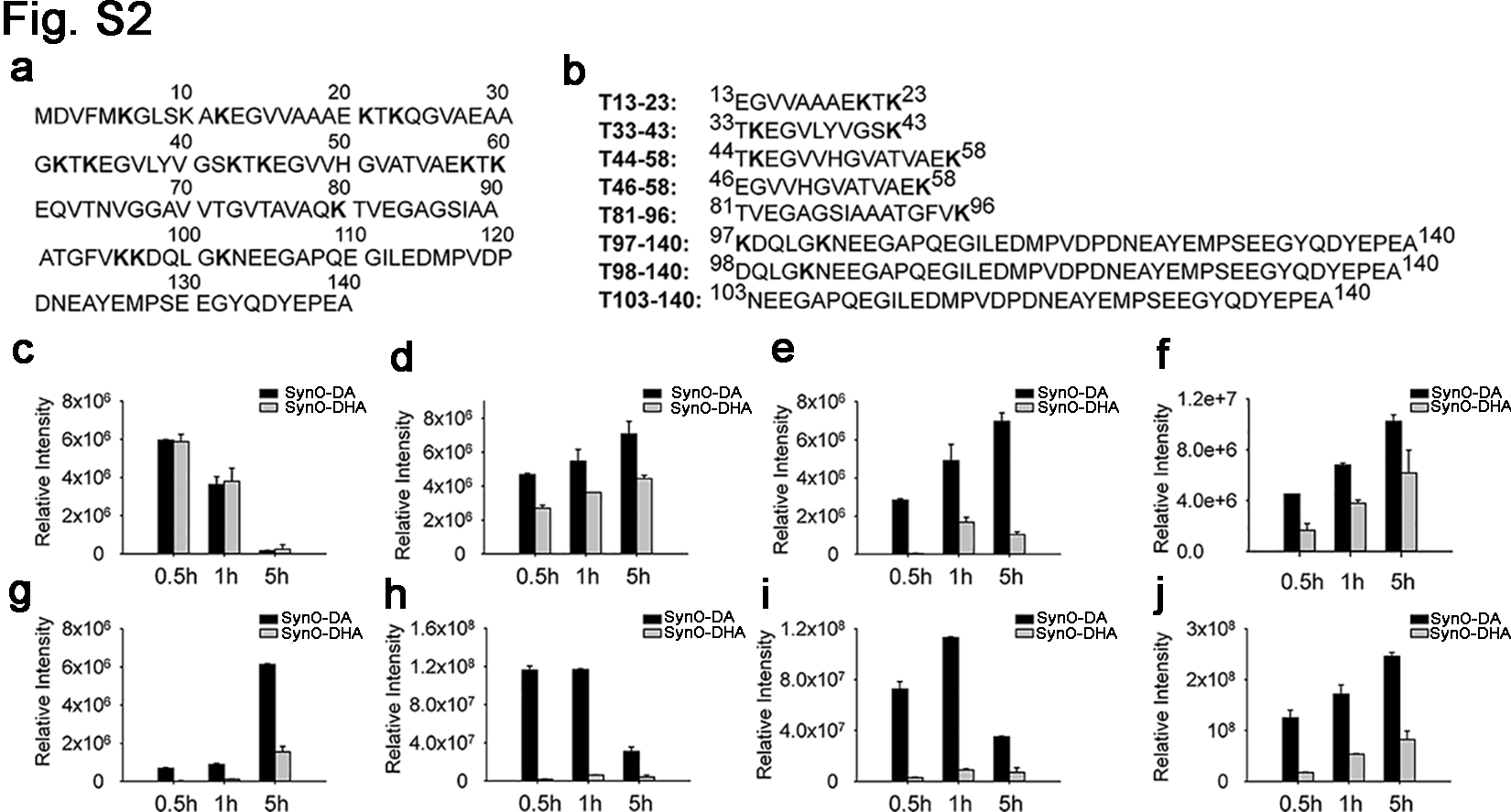

Supplement: Supplementary file 3 — Mass spectrometry (MS) analysis of trypsin digested fragments of the two α-Syn oligomeric polymorphs. SynO-DA and SynO-DHA were digested with trypsin for 0.5, 1 and 5 h under native condition. The resulting tryptic peptides were analyzed by mass spectrometry. The intensity of each peptide was normalized with the proteotypic peptide of α-Syn, EGVLYBGSK. (a) The amino acid sequence of α-Syn. (b) Tryptic peptides of SynO-DA and SynO-DHA that were analyzed by LC-MS. The relative intensities of tryptic peptides are shown: T13-23 (c), T33-43 (d), T44-58 (e), T46-58 (f), T81-96 (g), T97-140 (h), T98-140 (i), and T103-140 (j). (PNG 530 kb) [file 12035_2020_1913_Fig11_ESM.png]

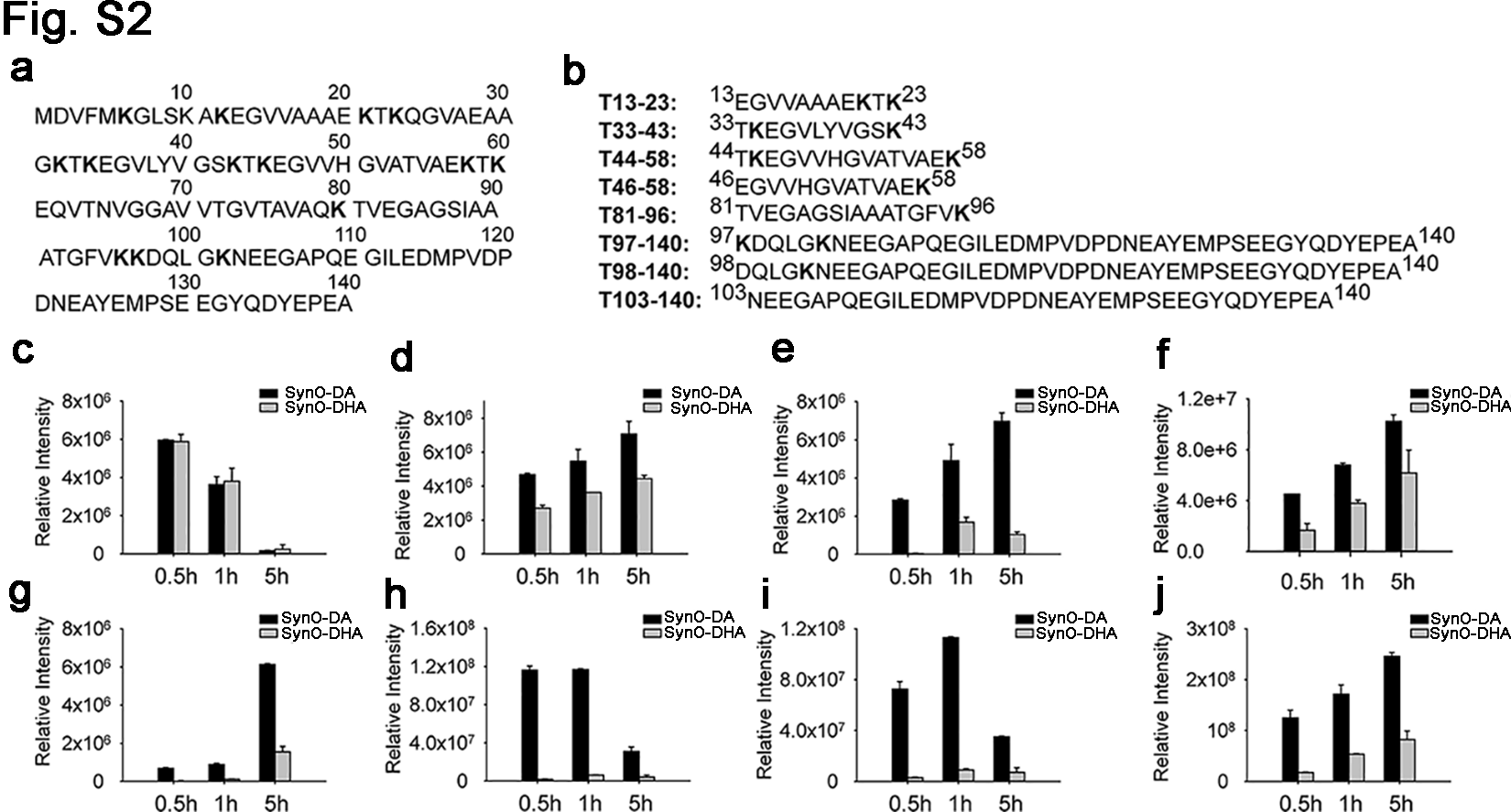

Supplement: Supplementary file 4 — High Resolution Image (TIF 432 kb) [file 12035_2020_1913_MOESM2_ESM.tif]

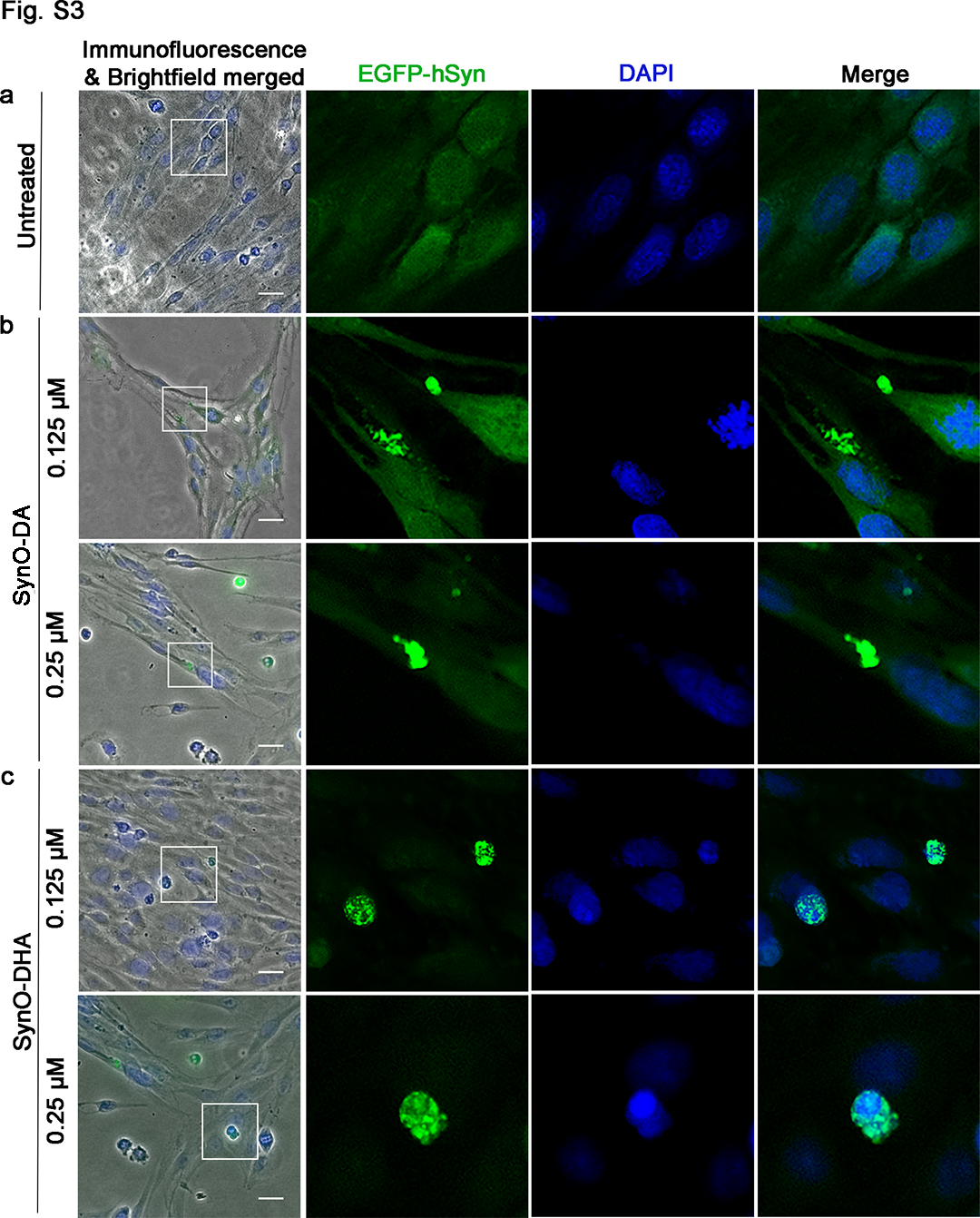

Supplement: Supplementary file 5 — Seeding potency of α-Syn oligomeric strains. (a-c) Representative brightfield and epifluorescence microscopic images of transiently EGFP-hSyn expressing SH-SY5Y cells exposed to SynO-DA and SynO-DHA at 0.125 and 0.25 μM concentrations for 16 h. Brightfield images merged with EGFP-hSyn (green) and DAPI (blue; nuclei) are shown on the left panels. Merged immunofluorescence images on right panels showed cytosolic α-Syn aggregates formed by the seeding with the different concentrations of α-Syn oligomeric strains: SynO-DA (b) and SynO-DHA (c). Scale bar 10 μm. (PNG 1446 kb) [file 12035_2020_1913_Fig12_ESM.png]

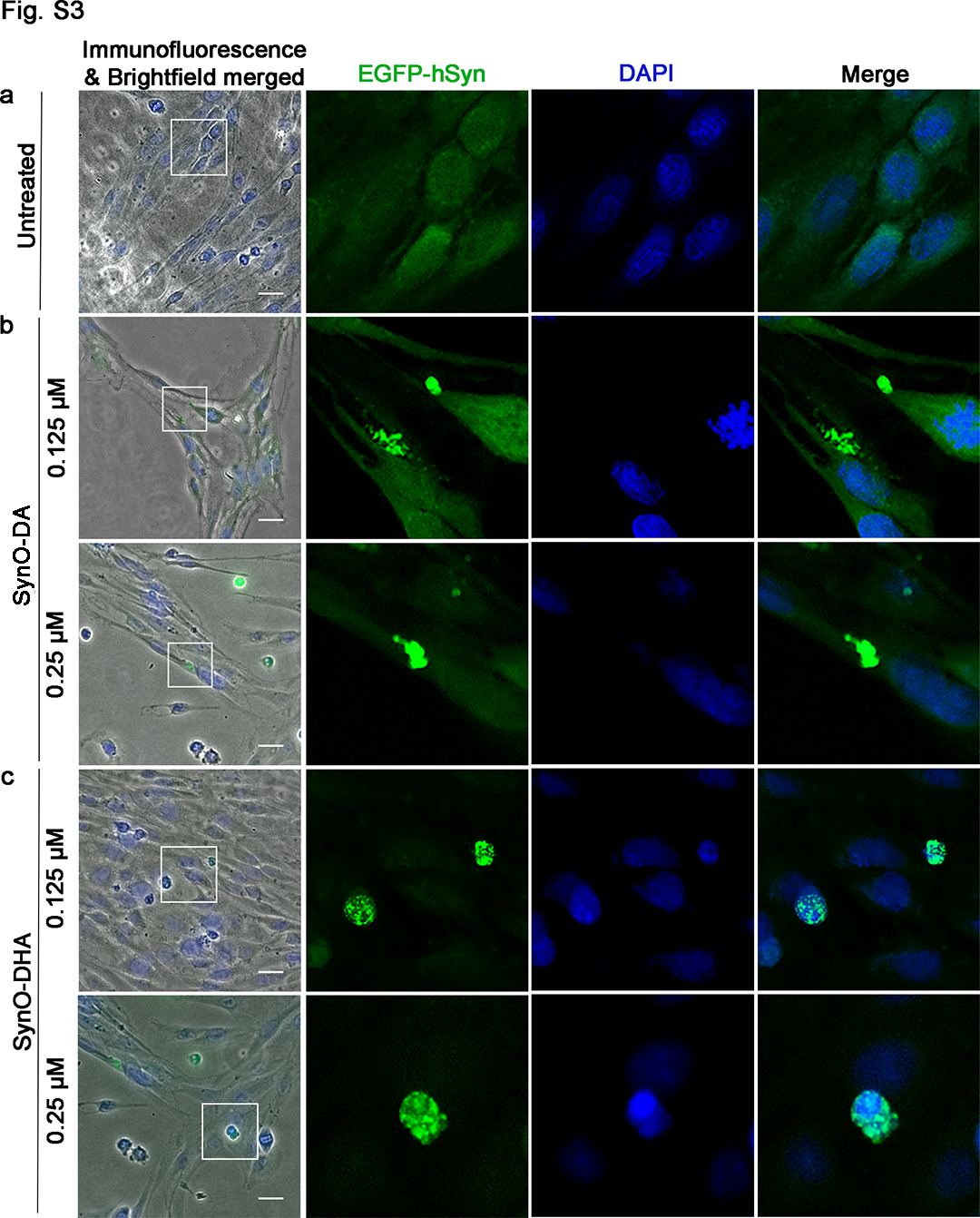

Supplement: Supplementary file 6 — High Resolution Image (TIF 1566 kb) [file 12035_2020_1913_MOESM3_ESM.tif]

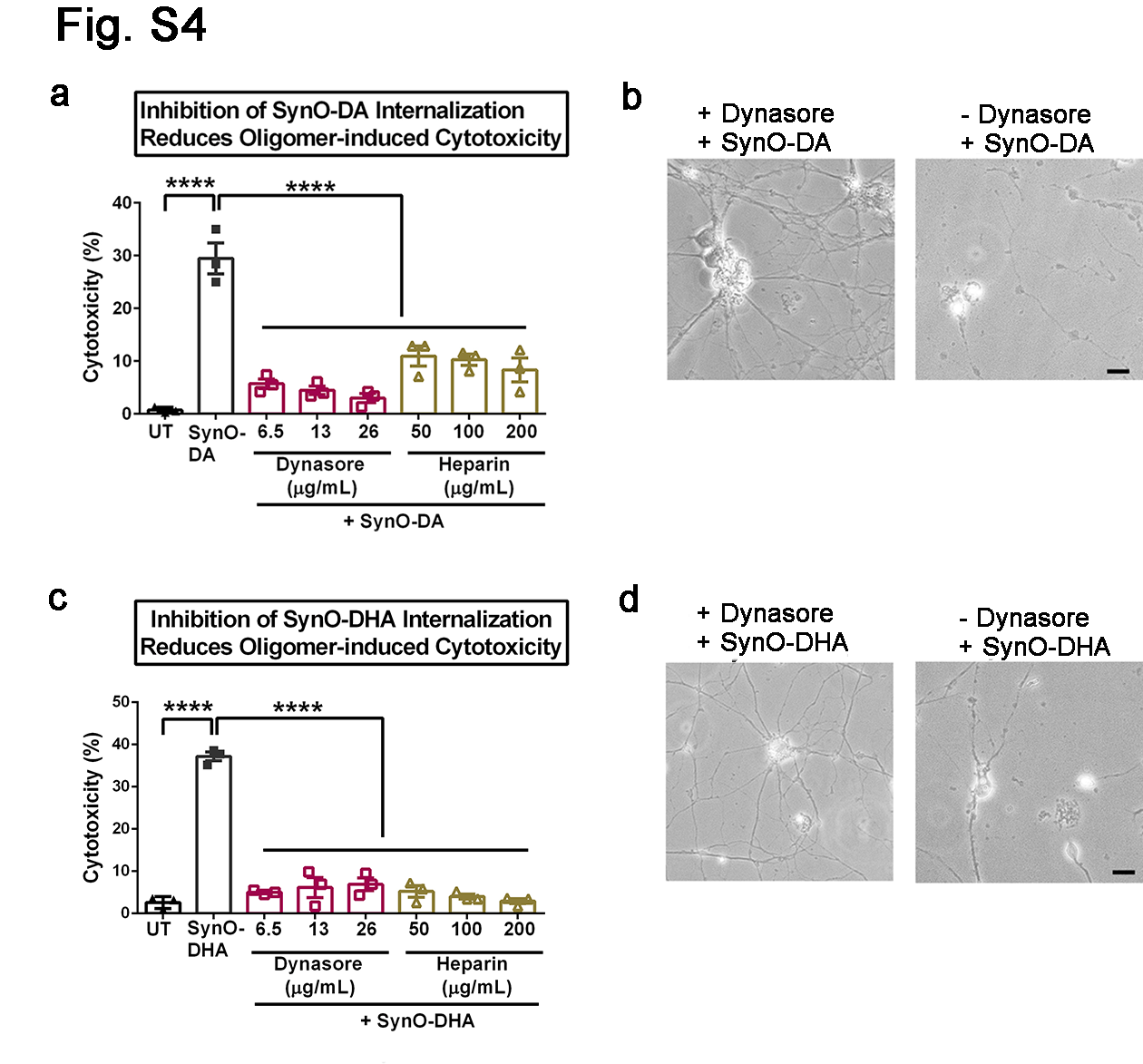

Supplement: Supplementary file 7 — HSPG and dynamin antagonists reduce α-Syn oligomeric strains internalization and cytotoxicity in neurons. Primary cortical neurons were pre-treated with three different concentrations of the two inhibitors: Dynasore (6.5-26 μg/mL) or Heparin (50-200 μg/mL) for 30 min. α-Syn oligomeric strains, SynO-DA and SynO-DHA were exogenously added to the cells at 1 μM concentrations and further incubated for a total of 16 h. (a, c) Cytotoxicity induced by SynO-DA (a) and SynO-DHA (c) in absence and presence of the two inhibitors was assessed by measuring LDH release. Internalization of oligomers was blocked in presence of both the inhibitors, thus rescuing oligomers induced toxicity. (b, d) Representative live cell images of the primary cortical neurons exposed to SynO-DA (b) and SynO-DHA (d) in presence and absence of the Dynasore inhibitor. Oligomer-induced toxicity was rescued when cells were treated in presence of Dynasore inhibitor. The quantification is represented as mean ± SD from three independent experiments. Statistical significance was calculated using one-way ANOVA with Tukey’s multiple comparison test, **** p<0.0001. Scale bar 10 μm. (PNG 502 kb) [file 12035_2020_1913_Fig13_ESM.png]

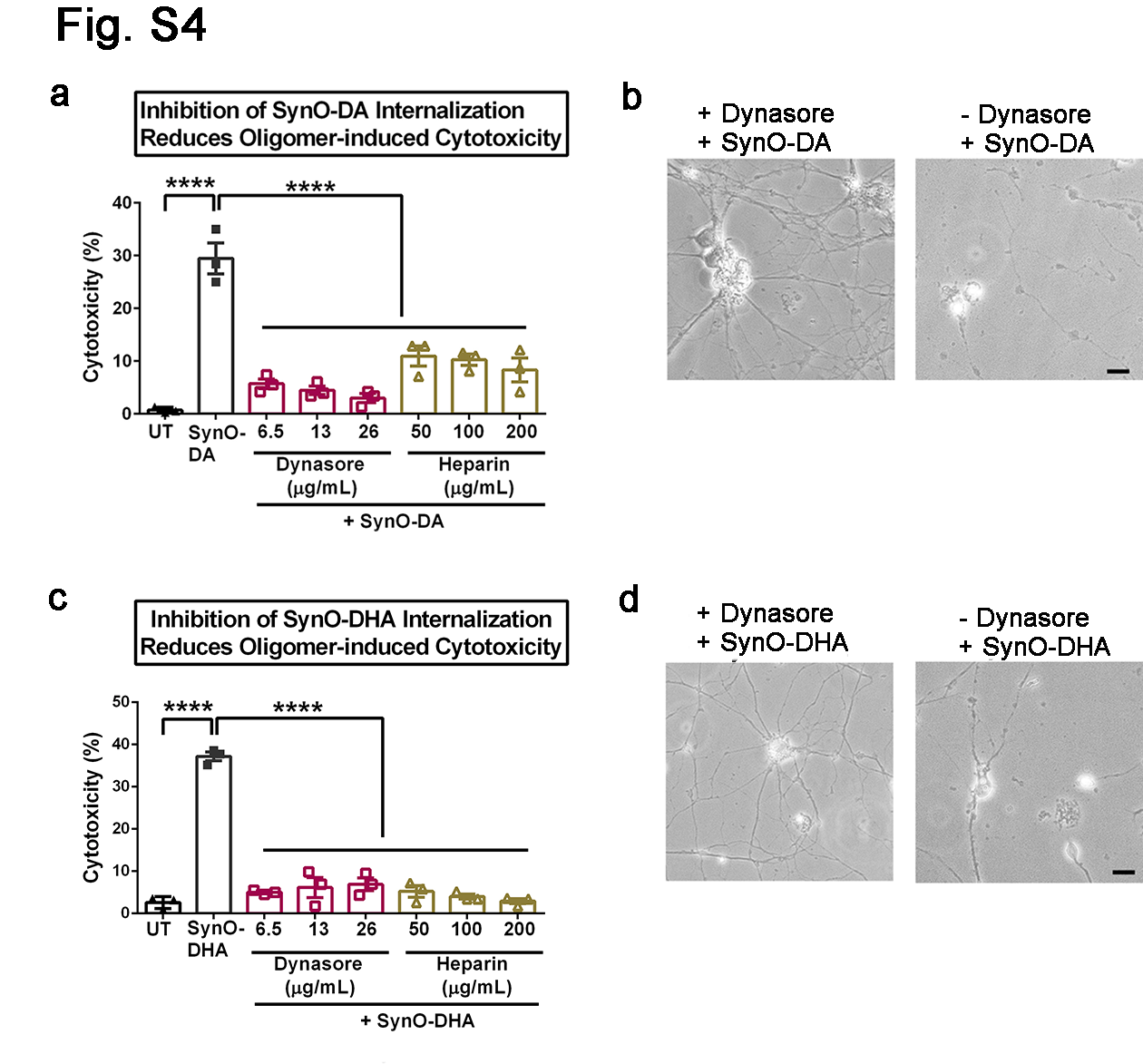

Supplement: Supplementary file 8 — High Resolution Image (TIF 532 kb) [file 12035_2020_1913_MOESM4_ESM.tif]

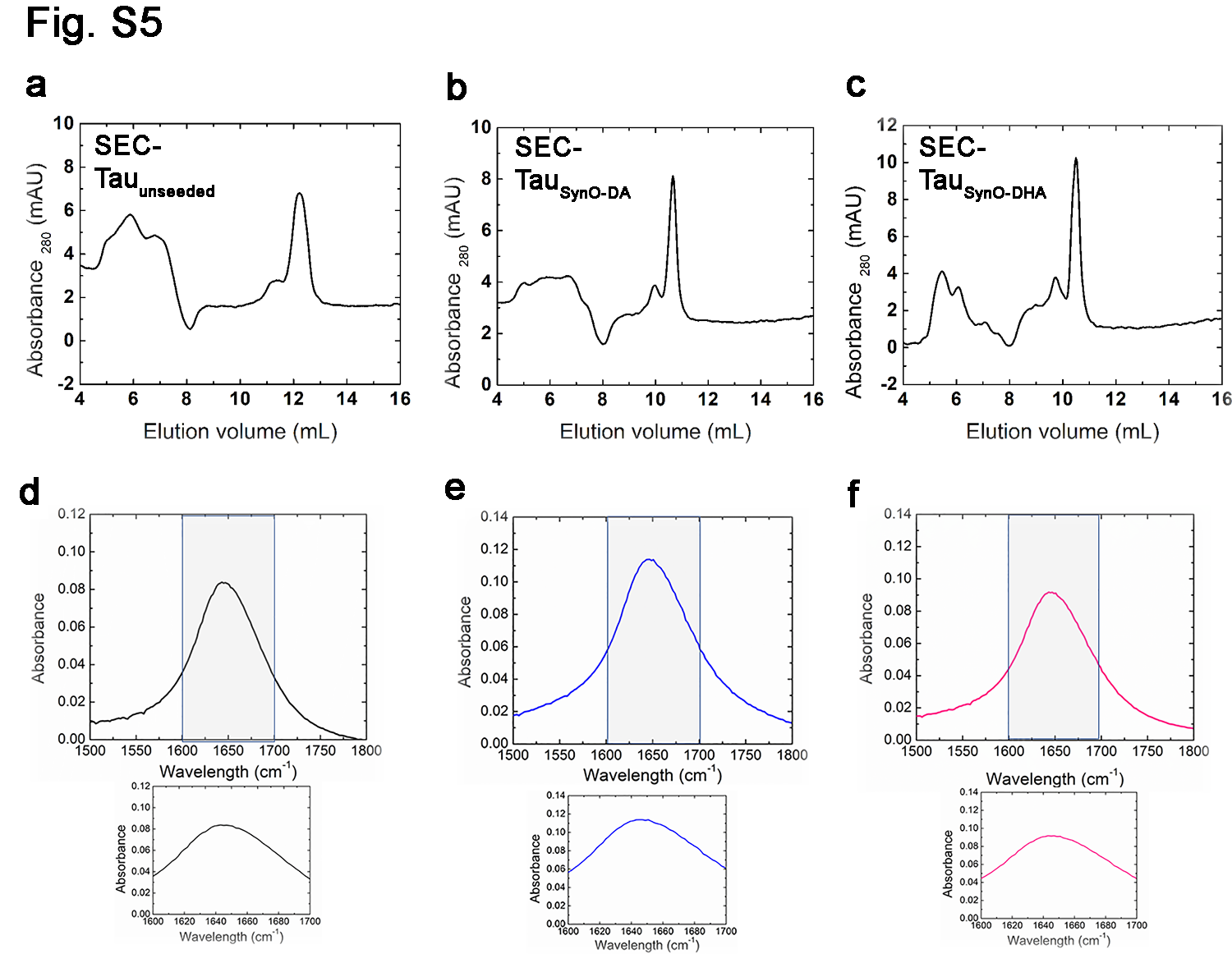

Supplement: Supplementary file 9 — Characterization of cross-seeded and unseeded tau aggregates. (a-c) Size exclusion chromatograms (SEC) showing peaks of different sizes of tau aggregates. (d-f) FTIR absorption spectra of all three tau aggregates with insets detailing the amide I region. (PNG 428 kb) [file 12035_2020_1913_Fig14_ESM.png]

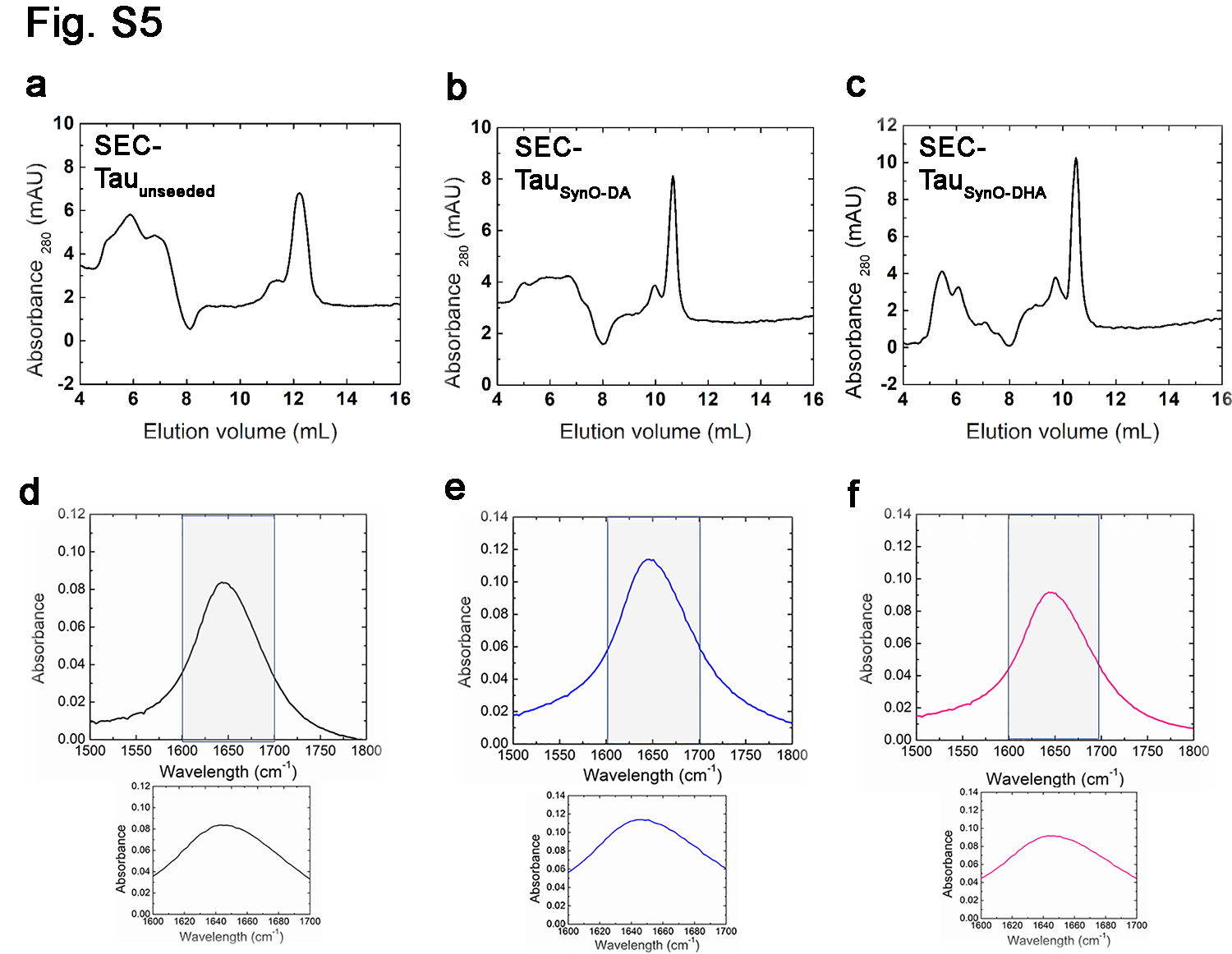

Supplement: Supplementary file 10 — High Resolution Image (TIF 486 kb) [file 12035_2020_1913_MOESM5_ESM.tif]

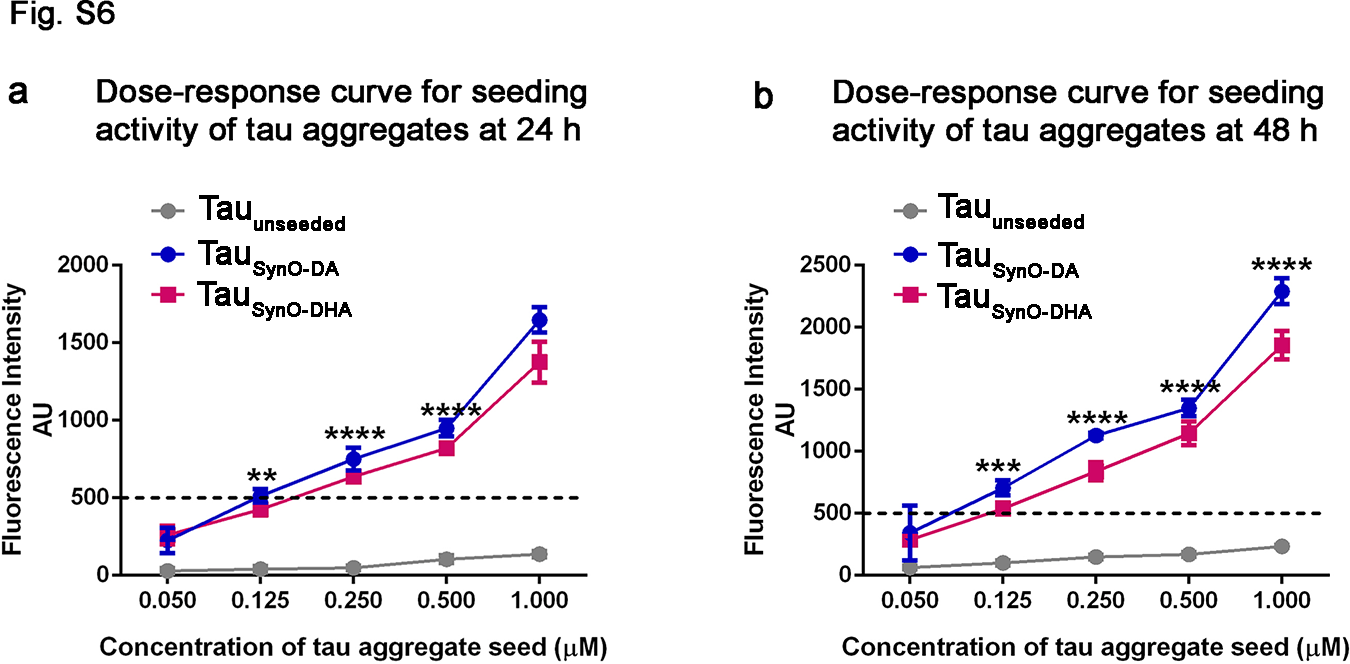

Supplement: Supplementary file 11 — Dose-response curves for seeding activity of tau aggregates. Tau biosensor cells were exposed to increased concentrations of the three tau aggregates (0.05, 0.125, 0.25, 0.5 and 1 μM) in presence of Lipofectamine and fluorescence intensity was measured at 24 h (a) and 48 h (b) time points. Data are represented as mean ± SD from four experimental replicates. Statistical significance was calculated using two-way ANOVA with Bonferroni post hoc analysis. ** p<0.01, *** p< 0.001, **** p<0.0001. (PNG 211 kb) [file 12035_2020_1913_Fig15_ESM.png]

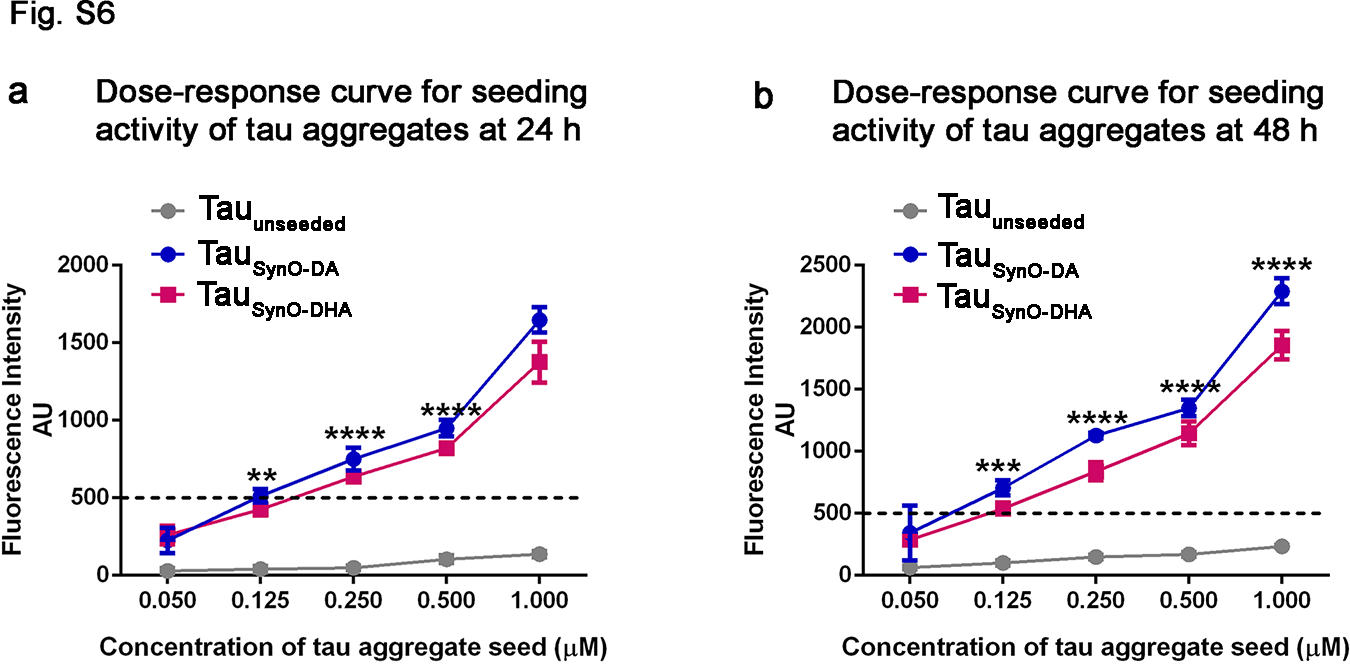

Supplement: Supplementary file 12 — High Resolution Image (TIF 223 kb) [file 12035_2020_1913_MOESM6_ESM.tif]
